# Comparison of diffusion tensor imaging by cardiovascular magnetic resonance and gadolinium enhanced 3D image intensity approaches to investigation of structural anisotropy in explanted rat hearts

**DOI:** 10.1186/s12968-015-0129-x

**Published:** 2015-04-29

**Authors:** Olivier Bernus, Aleksandra Radjenovic, Mark L Trew, Ian J LeGrice, Gregory B Sands, Derek R Magee, Bruce H Smaill, Stephen H Gilbert

**Affiliations:** Inserm U1045 - Centre de Recherche Cardio-Thoracique, L’Institut de rythmologie et modélisation cardiaque LIRYC, Université de Bordeaux, PTIB - campus Xavier Arnozan, Avenue du Haut Leveque, 33604 Pessac, France; Institute of Cardiovascular and Medical Sciences, University of Glasgow, BHF Glasgow Cardiovascular Research Centre, 126 University Place, Glasgow, G12 8TA UK; Auckland Bioengineering Institute, University of Auckland, Auckland, New Zealand; Department of Physiology, University of Auckland, Auckland, New Zealand; School of Computing, The University of Leeds, Leeds, LS2 9JT UK; Mathematical Cell Physiology, Max-Delbrück-Center for Molecular Medicine (MDC), Robert-Rössle-Straße 10, 13125 Berlin, Germany

**Keywords:** Diffusion tensor imaging, Cardiovascular magnetic resonance, Myocardium, Myolaminar

## Abstract

**Background:**

Cardiovascular magnetic resonance (CMR) can through the two methods 3D FLASH and diffusion tensor imaging (DTI) give complementary information on the local orientations of cardiomyocytes and their laminar arrays.

**Methods:**

Eight explanted rat hearts were perfused with Gd-DTPA contrast agent and fixative and imaged in a 9.4T magnet by two types of acquisition: 3D fast low angle shot (FLASH) imaging, voxels 50 × 50 × 50 μm, and 3D spin echo DTI with monopolar diffusion gradients of 3.6 ms duration at 11.5 ms separation, voxels 200 × 200 × 200 μm. The sensitivity of each approach to imaging parameters was explored.

**Results:**

The FLASH data showed laminar alignments of voxels with high signal, in keeping with the presumed predominance of contrast in the interstices between sheetlets. It was analysed, using structure-tensor (ST) analysis, to determine the most (**v**_**1**_^**ST**^), intermediate (**v**_**2**_^**ST**^) and least (**v**_**3**_^**ST**^) extended orthogonal directions of signal continuity. The DTI data was analysed to determine the most (**e**_**1**_^**DTI**^), intermediate (**e**_**2**_^**DTI**^) and least (**e**_**3**_^**DTI**^) orthogonal eigenvectors of extent of diffusion. The correspondence between the FLASH and DTI methods was measured and appraised. The most extended direction of FLASH signal (**v**_**1**_^**ST**^) agreed well with that of diffusion (**e**_**1**_^**DTI**^) throughout the left ventricle (representative discrepancy in the septum of 13.3 ± 6.7°: median ± absolute deviation) and both were in keeping with the expected local orientations of the long-axis of cardiomyocytes. However, the orientation of the least directions of FLASH signal continuity (**v**_**3**_^**ST**^) and diffusion (**e**_**3**_^**ST**^) showed greater discrepancies of up to 27.9 ± 17.4°. Both FLASH (**v**_**3**_^**ST**^) and DTI (**e**_**3**_^**DTI**^) where compared to directly measured laminar arrays in the FLASH images. For FLASH the discrepancy between the structure-tensor calculated **v**_**3**_^**ST**^ and the directly measured FLASH laminar array normal was of 9 ± 7° for the lateral wall and 7 ± 9° for the septum (median ± inter quartile range), and for DTI the discrepancy between the calculated **v**_**3**_^**DTI**^ and the directly measured FLASH laminar array normal was 22 ± 14° and 61 ± 53.4°. DTI was relatively insensitive to the number of diffusion directions and to time up to 72 hours post fixation, but was moderately affected by b-value (which was scaled by modifying diffusion gradient pulse strength with fixed gradient pulse separation). Optimal DTI parameters were b = 1000 mm/s^2^ and 12 diffusion directions. FLASH acquisitions were relatively insensitive to the image processing parameters explored.

**Conclusions:**

We show that ST analysis of FLASH is a useful and accurate tool in the measurement of cardiac microstructure. While both FLASH and the DTI approaches appear promising for mapping of the alignments of myocytes throughout myocardium, marked discrepancies between the cross myocyte anisotropies deduced from each method call for consideration of their respective limitations.

**Electronic supplementary material:**

The online version of this article (doi:10.1186/s12968-015-0129-x) contains supplementary material, which is available to authorized users.

## Glossary

**Myocyte orientation:** mean orientation of aggregated myocytes within a local spatial region

**Sheetlet:** localized sheet-like aggregations of myocytes ~ 6-cells thick extending as curved branching planes

**Sheetlet-interstices:** gaps between adjacent sheetlets which exist as potential spaces *in vivo* and open up on fixation. Collagen structure differs within sheetlets and adjacent to sheetlet interstices, and there is evidence that sheetlet-interstices function as shear layers *in vivo* [6]

**Myolaminar structure:** the combined structure formed by sheetlet and sheetlet-interstices

**Isotropic structure:** structure with properties (at any point) identical in all directions

**Anisotropic structure:** structure with properties (at any point) which are different dependent on direction

**Orthotropic structure:** structure with properties (at any point) which are different and can be described relative to a set of orthogonal perpendicular axes.

**Diffusion tensor imaging:** CMR of tissue anisotropy involving imaging the directionality and magnitude of water diffusion, which is represented as a mathematical tensor

**Structure tensor:** an image analysis mathematical tool (operator) which encodes directionality information from a standard image (2D or 3D) into a tensor.

## Background

Myocardial structure is important to cardiac electrical and mechanical function and alteration to this structure that accompanies disease can lead to important functional changes [1]. The ventricular myocardium is composed of continuously branching sheetlets of myocytes separated by sheetlet-interstices containing variable amounts of collagen. Importantly to the understanding of myocardial structure and function, it has been demonstrated in a series of studies that three principal orthogonal structural directions are present. These directions are: (i) along the local myocyte axis (**m**); (ii) perpendicular to the local myocyte axis in the sheetlet plane (**s**); and (iii) normal to the sheetlet plane (**n**) - a structural arrangement known as orthotropy [2-7]. It has been shown that myocardial mechanical properties and electrophysiological conductance are different along each axis [2,5,8-10]. The structure of the myocardium at a cellular level has been described in detail elsewhere [6,11]. Briefly, the myocardium consists of stacked branching myolaminae which are generally 4–6 cells (~80 -120 μm) thick [8,12]. The long-axes of the myocytes from which the myolaminae are composed have a regular organization being largely parallel to the epicardial surface and having the classically-described smooth ~120° transmural change in helix angle relative to the circumferential direction [8,13], often described as a helical arrangement. In the rat, myolaminar structure is present throughout the myocardium except in the sub-epicardium [6,11]. Within the myocardium there are regions of abrupt change in laminar orientation, such that the myolaminae have been described as belonging to two populations (reviewed previously [8]).

Measuring the orientations of these architectural features is important as they have roles in both electrophysiological and biomechanical function in health and disease. Changes in local myocyte orientation and myolaminar sliding (the shearing of adjacent myolaminae over each other) are thought to be the principal mechanisms of ventricular wall thickening in systole [8,9]. During contraction, force is generated along the local myocyte axis, and local myocyte orientation has long been known to influence the spread of myocardial activation [14], which has recently been shown to be substantially influenced by laminar organization also [2,5]. Knowledge of local myocyte and laminar architecture is therefore important in the understanding of normal cardiac function, in the interpretation of electrical and mechanical studies of cardiac disease in animal models, and, in the long term, may be relevant to the interpretation of clinical cardiac electrophysiology and mechanical recording/imaging. In addition, whole-heart computational modeling of both mechanics and electrophysiology requires detailed structural atlases and Diffusion Tensor Magnetic Resonance Imaging (DTI) is the principal method for generating these geometries [15-17].

Histological validation studies have shown that DTI can be used to measure cardiac local myocyte orientation [18]. Histological validation is experimentally challenging for two reasons: (i) the orthotropic structure of the heart often confuses interpretation of 2-dimensional structural images, and, (ii) the orientation of cardiomyocytes within a local region is an abstract concept where there are no myocardial *fibers* in the true sense, only myocytes, with multiple branching, and a maximum length of ~120 μm (discussed in [19]). Indeed this difficulty was recognized by authors of early validation studies [18]. Later, it was proposed that DTI could be used as a 3-dimensional method to measure myocardial laminar orientations [20,21], and fully quantify ventricular myocardial orthotropy. This was an important claim as, if correct, DTI could deliver, from a single imaging experiment, a description of cardiac orthotropy which can be directly used for the computational modeling of cardiac electrophysiology and mechanics. Early validation of DTI laminar structure measurement used non-conventional approaches for the study of tissue architecture (the paper ink blotting of dead tissue) [21]. However, soon after DTI orthotropy measurement was proposed questions and challenges were raised in the literature (from detailed physical studies) about the appropriateness of DTI for measuring laminar orientation (and to a lesser extent local myocyte orientation) [22,23].

A new method for directly imaging myocardial laminar architecture is high-resolution 3D FLASH CMR (previously referred to as high resolution CMR [11]), which was introduced by [24] (using T2* contrast). Here the term high-resolution was used with respect to the whole heart geometry and with respect to clinical cardiovascular magnetic resonance (CMR), not with respect to the myocyte/sheetlet dimensions. We recently developed this method further (using Gd-DTPA T1 contrast) and validated it as a means to measure whole ventricular 3D myolaminar architecture [11]. We showed that myolaminar architecture could be imaged and measured using FLASH and that the orientations, obtained using a well-known mathematical operator, the structure tensor (ST), corresponded to histologically measured orientations [11]. This method analyses a tensorial quantity constructed from the structure of the image, very much like the DTI tensor [25-28]. From the mathematics of the ST method, its primary eigenvector is in the direction of maximum image contrast change, and the tertiary eigenvector in the direction of minimum image contrast change. Throughout this manuscript we implement a notation of **v**_**1**_^**ST**^, **v**_**2**_^**ST**^ and **v**_**3**_^**ST**^ for the ST eigenvectors to simplify the comparison between ST and DTI measurements. In this notation **v**_**1**_^**ST**^ relates to the tertiary ST eigenvector, **v**_**2**_^**ST**^ relates to the intermediate ST eigenvector and **v**_**3**_^**ST**^ relates to the primary ST eigenvector. As the myocardium has an orthotropic structure we hypothesize that the primary eigenvector is the sheetlet normal direction; the secondary eigenvector the sheetlet in-plane direction and the tertiary eigenvector the local myocyte direction.

The aim of this study is to compare the structural measurements from the DTI and ST/FLASH, to demonstrate how both these measures relate to the laminar structure as directly imaged (the laminar structure as revealed by FLASH) and to consider the potential strengths, limitations and applications of these approaches. Our hypothesis is that the myolaminar orientations provided by the FLASH 3D ST would be more accurate and reliable than those measured by DTI, as the primary ST eigenvector (the largest) is calculated from the sheetlet normal direction, and the approach is not subject to the limitations of the DTI model concerning multiple diffusion compartments.

In our analysis we refer to the true orientations of the myocyte, sheetlet-plane and sheetlet normal directions as **m**, **s**, and **n** respectively. When referring to image measured structural orientations and derived structural angles we use the notation for the DTI eigenvectors (**e**_**1**_^**DTI**^, **e**_**2**_^**DTI**^, **e**_**3**_^**DTI**^) and for the ST derived orthogonal vectors (**v**_**1**_^**ST**^, **v**_**2**_^**ST**^, **v**_**3**_^**ST**^) until we establish association between the eigenvector and the structural feature. When we refer to the structural feature directly we use the term *putative* to indicate that this association is not yet confirmed.

## Methods

### Heart preparation and perfusion fixation

Male Wistar rats (N = 8) weighing 220.1 ± 11.2 g were euthanized in accordance with the UK Home Office Animals (Scientific Procedures) Act 1986 with the approval of the UK Home Office and the Local Ethics Committee. Hearts were rapidly dissected, the aorta cannulated and the hearts perfused in Langendorff mode with CMR contrast agent (Gd-DTPA) and fixative for 20 min. Details are given in the Methods Supplement (Additional file 1) and are as described in [11]. The hearts were then removed from the perfusion apparatus and stored 2 hours at 20°C in the contrast/fixative solution before imaging.

### FLASH Acquisition

The imaging order and parameters are summarized in Table 1. FLASH was carried out using a T1-weighted (T1W) FLASH (Fast Low Angle SHot) CMR sequence in a Bruker (Ettlingen, Germany) 9.4T CMR scanner with 20 averages and echo time (TE) = 7.9 ms, repetition time (TR) = 50 ms, with 20 averages taking 18 h to acquire at a resolution of 50 × 50 × 50 μm^3^ at 20°C.

**Table 1:** **Summary of the imaging sequence applied for sensitivity analysis**

**Ref#**	**Imaging Parameters**				
	**Start time** ^**a**^	**Type**	**n-dir**	**b-value (s /mm** ^**2**^ **)**	**Scan duration** ^**b**^
**1**	2:00	DTI	6	1000	1:50
**2**	3 :50	DTI	12	1000	3:56
**3**	7:46	DTI	6	1000	1:50
**4**	9:36	DTI	12	500	3:56
**5**	13:32	DTI	6	1000	1:50
**6**	15:22	DTI	12	2000	3:56
**7**	19 :18	DTI	6	1000	1:50
**8**	21:08	T1W	NA	NA	18:12
**9**	39:20	DTI	6	1000	1:50
**10**	41:10	DTI	12	1500	3:56
**11**	45:06	DTI	6	1000	1:50
**12**	46:57	T1W	NA	NA	18:12
**13**	65:09	DTI	6	1000	1:50
**14**	66:59	DTI	12	2500	3:56
**15**	70:55	DTI	6	1000	1:50

The first scan was carried out at 2 h and subsequent serial scans were carried out without moving the heart in the scanner. A baseline scan taking 1 h 50 m for 6 diffusion directions at b = 1000 s:mm^2^ was performed at serial time-points throughout the imaging study to explore any changes in diffusion as a result of time elapsed from killing/perfusion fixation. Interspersed between these baseline scans two T1W FLASH scans were performed at 50 × 50 × 50 μm resolution, as well as a series of diffusion scans with different numbers of directions and different b-values. All diffusion scans were at 200 × 200 × 200 μm resolution. Ref #: reference number for this scan; ^a^– time post-fixation (h:min); ^b^duration (h:min); Type: DTI - diffusion tensor magnetic resonance imaging; T1W is T1-weighted FLASH; FLASH: fast low angle shot; n-dir: number of diffusion directions; NA: not applicable.

### DTI Acquisition and Reconstruction

**Figure 1:**
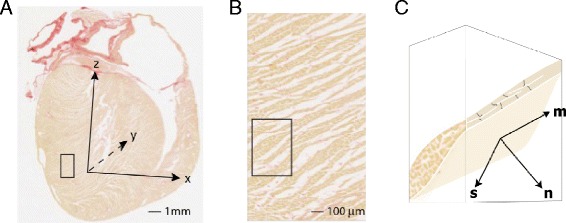
**Local structure-based coordinate system. A** - long axis histological section from rat heart indicating the cardiac coordinate system (x,y,z). The z axis passes through the left ventricle apex and the center of the mitral valve orifice. **B** - magnified view of the region identified by box in (A). **C** - schematic 3D representation of a single layer of myocytes within the sub-region indicated in (B). This representation is simplified to facilitate labelling of the local structure-based coordinate system. The lamina consists of branching myocytes and is bounded by a network of perimysial collagen. In the local axis system, **m** aligns with the myocyte axis, **n** is normal to surface of the lamina and **s** is orthogonal to **m** and **n**. This cartoon does not show important microstructural features. These include i) branching and interconnection of laminae ii) curvature of laminae and myocyte orientation, and iii) the existence of adjacent laminae with different orientations.

DTI was carried out using the same MR scanner at 20°C with a resolution of 200 × 200 × 200 μm^3^ and using a 3D diffusion-weighted simple spin-echo sequence with reduced encoding and with TE (echo time) = 15 ms, TR (recovery time) = 500 ms, field of view 12.8 × 12.8 × 25.6 mm^3^, matrix 64 × 64 × 128. Diffusion gradients were monopolar and had 3.6 ms duration and 11.5 ms separation. The heart was not moved in the scanner between imaging studies. In order to carry out a sensitivity analysis of DTI laminar measurement a series of imaging experiments were carried out with changing b-value (in the range 500–2500 s/mm^2^, with the b-value scaled by changing the diffusion gradient pulse strength at constant pulse separation) and two sets of optimized gradient directions (6 and 12) [29] (Table 1). The first baseline study (scan 1 in Table 1) had 6 directions (plus the b0 direction) which is the minimum number of directions from which the diffusion tensor can be calculated. In order to carry out a sensitivity analysis one parameter was changed from this sequence for a series of subsequent imaging experiments. As the time post-fixation also inevitably changes, this baseline sequence was repeated after each later scan, so that sensitivity to time post-fixation could be explored. Reconstruction of the raw diffusion weighted images to the diffusion tensor, calculation of the eigenvectors and eigenvalues and calculation of the derived myocyte and sheetlet orientation has been described previously [30]. For each scan a single b0 image (b-value = 0 s/mm^2^) was acquired and processing of the raw diffusion weighted images to the diffusion tensor was carried out using ParaVision 4.2 (Bruker GmbH, Ettlingen, Germany). The eigenvector corresponding to the largest magnitude eigenvalue (**e**_**1**_^**DTI**^) is the putative local myocyte orientation, the eigenvector corresponding to the intermediate magnitude eigenvalue (**e**_**2**_^**DTI**^) is the putative sheetlet/laminae in-plane orientation and the eigenvector corresponding to the smallest magnitude eigenvalue (**e**_**3**_^**DTI**^) is the putative sheetlet/laminae normal direction (Figure 1, Table 2).

**Table 2:** **Summary of the notation used for vectors and angles**

**e**_**1**_^**DTI**^, **e**_**2**_^**DTI**^ ** and e**_**3**_^**DTI**^	DTI eigenvector corresponding to the most, intermediate and least extended directions of diffusion
**v**_**1**_^**ST**^, **v**_**2**_^**ST**,^ **v**_**3**_^**ST**^	the vector of the most, intermediate and least extended orthogonal directions of FLASH signal, determined by structure-tensor analysis.
λ_1_^ST^	eigenvalue (λ) with the subscript indicating the eigenvalue number.
α'**v**_**1**_^ST^ and α'**e**_**1**_^DTI^	vector helix angle, used for quantification of the putative myocyte orientation. The vector quantified is identified after α’. The angle is defined in Figure 2.
α''**v**_**1**_^ST^ and α''**e**_**1**_^DTI^	vector transverse angle, used for quantification of the putative myocyte orientation.
β'**v**_**3**_^ST^ and β'**e**_**3**_^DTI^	vector elevation angle, used for quantification of the putative sheetlet/sheetlet-normal orientation.
β”**v**_**3**_^ST^ and β''**e**_**3**_^DTI^	vector transverse angle, used for quantification of the putative sheetlet/sheetlet-normal orientation.
**m**	the true myocyte orientation vector.
**s**	the true sheetlet (in-plane) vector.
**n**	the true sheetlet normal vector. The superscript FI is used in the case of **n** measured by FLASH/FI.

**Figure 2:**
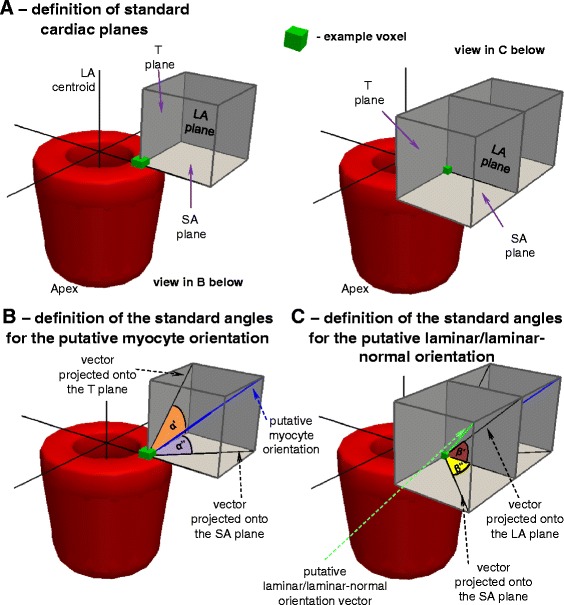
**Definitions of the myocyte and sheet angles with respect to the standard cardiac planes. A** – definitions of the three standard cardiac planes. **B** – definitions of the myocyte angles. **C** – definitions of the sheet angles. **D** - definitions of the sheet normal angles. LA: long-axis; SA: short-axis; T: tangent. The symbols for vectors and derived angles are defined in Table 2.

The angles, cardiac reference planes and cardiac coordinate system are defined in Figure 1 and Figure 2 Note: the putative myocyte helix-angle α’ is projected onto the wall-tangent plane whereas the β’ elevation angles associated with the putative sheetlet in-plane and normal vectors **(s** and **n)** are projected onto the long-axis plane. FLASH: fast low angle shot; ST: structure tensor of FLASH data; DTI: diffusion tensor magnetic resonance imaging.

### Structure tensor analysis of high resolution MR images

The following steps were applied to the FLASH images: segmentation, conversion to a stack, boundary smoothing, intensity gradient computation, structure tensor calculation for each voxel, and extraction of principal directions of the structure tensor at each discrete point using eigenanalysis. In detail, the FLASH images were coarsely segmented to remove the ventricular cavity signal using thresholding and semi-automated segmentation in Seg3D (Scientific Computing and Imaging Institute, University of Utah). These FLASH images were then converted to a stack (256 × 256 × 512) of 16-bit images. To avoid undue influence on structural orientation calculations, boundaries at the interface between tissue and non-tissue regions in the CMR images were smoothed as described in [27]. Myostructural orientations were computed from the images by computing intensity gradients with a 3 × 3 × 3 or 5 × 5 × 5 point derivative template [26] (the derivative template width, DTW). The template was applied to the full 3D image using FFT-based convolution. The structure tensor (the outer product of the intensity gradient vectors) was then computed for each voxel in the 3D image. Structure tensor components at progressive resolution doubling (i.e. 100 μm, 200 μm, 400 μm, etc.) were determined using level 2 or level 4 binomial low-pass filters [31] to smooth from one level of resolution to the next. The smoothing template width (STW) for these two configurations was 3 and 5 points, respectively. The 200 μm smoothed structure tensor data set (64 × 64 × 128 tensors) was used in order to best match the DTI resolution. The principal directions of the structure tensor at each discrete point were extracted using eigenanalysis. The eigenvector corresponding to the largest magnitude eigenvalue is the least extended orthogonal direction of signal continuity and is therefore the putative sheetlet/laminae normal direction, and for ease of comparison with DTI this vector is denoted by **v**_**3**_^**ST**^. The eigenvector corresponding to the smallest magnitude structure tensor eigenvalue is the most extended orthogonal direction of signal continuity and is therefore the putative local myocyte-orientation, and for ease of comparison with DTI this vector is denoted by **v**_**1**_^**ST**^. The eigenvector corresponding to the intermediate magnitude eigenvalue is the intermediate extended orthogonal direction of signal continuity and is therefore the putative sheetlet/laminae in-plane direction, and for ease of comparison with DTI this vector is denoted by **v**_**2**_^**ST**^. Vectors computed at points lying in non-tissue regions of the image were discarded on the basis of an automated fine-detail 8-bit tissue mask created slice-wise from the segmented images by thresholding intensity values (≤20% intensity) and performing the following sequence of morphological operations: (i) clean (removing isolated foreground pixels); (ii) bridge (connecting pixels separated by one background pixel); (iii) fill (filling isolated background pixels); (iv) open (binary opening); and (v) thicken (adding pixels around the exterior of an object without connecting previously unconnected pixels).

### Assignment of the cardiac reference frame

A prolate spheroidal coordinate frame was used, as has commonly been applied in the literature [32]. The framework for assignment of the cardiac reference frame was described in [27]. In order to automatically and without bias select regions of interest (ROI) for quantification, a model cardiac geometry with a manually fitted left ventricle long-axis centroid was registered to each heart CMR. Four cuboidal transmural equatorial ROI were defined in the model geometry: lateral, anterior, septal and posterior. The lateral, anterior and posterior ROI span from endocardium to epicardium and the septal ROI spans from left ventricular septal endocardium to right ventricular septal endocardium. This was carried out with Insight Tool Kit [33] using fast affine registration (as implemented in Slicer3, www.slicer.org) with 30 histogram bins, 40000 spatial samples and 400 iterations. The registered model hence defines: (i) the long-axis (LA) centroid of a cylindrical coordinate system for which the helix and transverse-angles of the eigenvectors were calculated; and, (ii) selected ROI for quantification. This registration defines precisely the same ROI and left ventricle long axis for the DTI data and for the FLASH data, and does not result in any transformation or deformation of the original DTI or FLASH/ST images. A pair of orientation angles (an angle of elevation and a corresponding transverse angle) are reported for each of the putative structural vectors (**v**_**1**_^**ST**^, **v**_**2**_^**ST**^, **v**_**3**_^**ST**^, **e**_**1**_^**DTI**^, **e**_**2**_^**DTI**^ and **e**_**3**_^**DTI**^). In detail, all three putative elevation angle pairs from FLASH/ST and DTI (α’**v**_**1**_^**ST**^, α’**e**_**1**_^**DTI**^; β’**v**_**2**_^**ST**^, β’**e**_**2**_^**DTI**^; β’**v**_**3**_^**ST**^, β’**e**_**3**_^**DTI**^) are measured with respect to the cardiac short-axis plane. Putative myocyte transverse angles (α”**v**_**1**_^**ST**^, α”**e**_**1**_^**DTI**^) are measured with respect to projections onto the wall-tangent plane (sometimes known as the circumferential-longitudinal plane), as myocytes run approximately parallel to this plane. Putative sheet (and sheet-normal) transverse angles (β”**v**_**2**_^**ST**^, β”**e**_**2**_^**DTI**^ ; β”**v**_**3**_^**ST**^, β”**e**_**3**_^**DTI**^) are measured with respect to projections onto the long-axis plane (sometimes known as the radial-circumferential plane) following conventions in the literature [8,11,30,34]. The planes and angles are illustrated in Figure 2. The local myocyte helix angle (α’) is the angle between the short-axis plane and the projection of the putative myocyte-orientation vector onto the wall-tangent plane. The local myocyte transverse angle (α”) is the angle between the wall-tangent plane and the projection of the putative myocyte vector onto the short-axis plane. The angle between the short-axis plane and the projection of the putative laminar in-plane vector onto the long-axis plane is β’. The angle between the longitudinal—radial plane and the projection of the laminar in-plane vector onto the short-axis plane is β”. The angles of orientation reported for the laminar normal correspond to the angles reported for the laminar in-plane vector, with the eigenvector analyzed named with each β’ and β” angle reported. Note, the sheetlet angles reported here are the angles with respect to the sheetlet vectors (in-plane or normal) projected onto the long-axis planes. These are sometimes known as *apparent* sheetlet angles and differ from the *absolute* sheetlet angle which is between the radial axis and the vector lying in the sheet plane (known variously as β or β^s^ [8,35].

### Comparison of structure tensor and diffusion tensor orientations

The ST data was smoothed to the resolution of the DTI data (from 50 μm 256 × 256 × 512 tensors to 200 μm 64 × 64 × 128 tensors). Systematic comparison of the ST and DTI is facilitated as images are in the same CMR frame/position (the hearts were not moved in the scanner between FLASH/ST and DTI). Comparison between different hearts is achieved through the automated approach for finding the left ventricle long-axis and ROI. For each ROI the angles between the eigenvectors and the helix and transverse angles were quantitatively compared.

### Comparison of structure tensor and diffusion tensor sheetlet orientations to the FLASH isosurface

**Figure 3:**
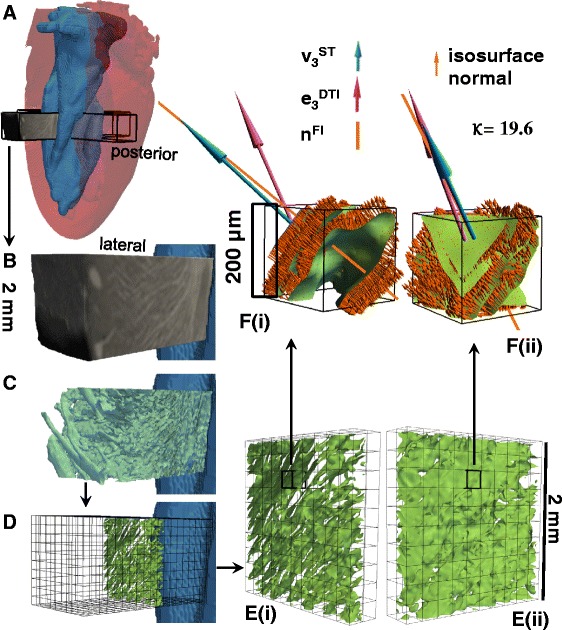
**Interactive segmentation of laminar architecture applied to all 200 μm cubes in the four ROIs to assess ST and DTI performance against the FLASH image from visualization based direct measurement. A** – the location of the ROI is shown in the transparent whole heart volume seen from the posterio-lateral view. **B** – the cropped unprocessed FLASH image of the lateral ROI from the same posterio-lateral view; **C** – a contour (the green isosurface) was generated in the lateral ROI of the raw CMR image at a specified intensity threshold value (after upsampling/interpolating), the contour delineates the boundary between myolaminae and sheetlet interstices (except in the sub-epicardium, where laminae are absent) – the contour is shown within the CMR raw image; **D** - the contour has been divided into cubes of 200 × 200 x 200 μm^3^, and each cube corresponds to one DTI or ST voxel (the grid of these cubes is shown for the whole lateral ROI from a posterio-lateral view); **E** – the DTI/ST voxel cubes are shown, from a posterio-lateral view in **(i)** and from an anterio-lateral view in **(ii)**, for the single transmural distance; **F** – a magnified view is shown of the previously highlighted individual voxel from **(E)**, and on the laminar contour the normal to the contour is shown by the multiple small orange arrows, with the mean FLASH laminar orientation in this 200 μm cube (FI, **n** ^**FI**^) shown by the orange line, the ST normal vector (**v**_**3**_^**ST**^) in blue, and the DTI normal vector in purple (**e**_**3**_^**DTI**^). It can be seen that the highlighted voxel that **v**_**3**_^**ST**^ is closer than **e**_**3**_^**DTI**^ to **n** ^**FI**^ (7.8° between **v**_**3**_^**ST**^ and **n** ^**FI**^, 24.7° between **e**_**3**_^**DTI**^ and **n** ^**FI**^, **K** = 19.6). DTI: Scan #1, 6-direction, b = 1000 s/mm^2^; ST: Scan #8; DTW = 3, STW = 3. FLASH: fast low angle shot; ST: structure tensor of FLASH data; DTI: diffusion tensor magnetic resonance imaging; FI: FLASH isosurface data; DTW: derivative template width STW: smoothing template width. The symbols for vectors and derived angles are defined in Table 2.

The DTI and ST sheetlet-normal orientation was qualitatively and quantitatively compared to interactive measurement of laminar normal orientation from isosurfacing of the FLASH images, as illustrated in Figure 3 (here known as the FLASH Isosurface, FI) The optimal threshold delineating sheetlets from sheetlet-interstices was determined by examination of the images, and this boundary was then generated into an isosurface consisting of a finite element surface mesh. This method, unlike DTI and ST/FLASH allows discrimination of two or more sheetlet orientations in a single 200 μm isotropic voxel, however, for the purpose of assessing the DTI and FLASH/ST methods we only consider those isosurfaced 200 μm isotropic voxels which contain a single sheetlet orientation. Although this method is slow and computationally and manually intensive to determine, it is highly robust and can be directly related to the observed FLASH structure. This was carried out in the lateral, anterior, septal and posterior cuboidal ROI. In detail, the raw FLASH data was upsampled 4 × using cubic regression interpolation from 50 μm isotropic voxels to 12.5 μm isotropic voxels (in Seg3D). A contour (isosurface) was then generated based on an appropriate CMR intensity threshold chosen from the myolaminae and sheetlet-interstices signal intensities. The contour defines the boundary between myolaminae and sheetlet-interstices and is a computational finite element surface (i.e. a surface made up of numerous linked equilateral triangles, each with a defined normal vector). The contour in the ROI was then separated into 200 μm boxes (which correspond exactly to the native voxels of the DTI imaging sequence and of the ST, here called DTI-ST-boxes). Within each DTI-ST-box the circular (axial) mean normal vector to the contour surface was determined through averaging of the finite element surface normals, and is referred to as **n**^**FI**^ (normal to the FLASH isosurface). As the laminar architecture is a branching network, some 200 μm DTI-ST-boxes contain branching, highly complex or multiple laminar orientations, or fall entirely on sheetlet-interstices or on myolaminae with little interface surface, for which it is not possible to describe a single laminar orientation, and the circular (axial) mean normal to the contour is therefore not a useful measure. These 200 μm DTI-ST-boxes were excluded based on a threshold of the concentration parameter (**K**, Kappa) of the spherical form of the von Mises-Fisher probability distribution [36]. The greater the value of **K** the greater the concentration of the normals around the mean orientation, and hence the more simple the laminar architecture of the 200 μm cube. The threshold applied (**K** = 7.0, i.e. **K**^**−1**^ = 0.143) was determined from sample 200 μm DTI-ST-boxes which inspection showed to have a simple laminar architecture. The **n**^**FI**^ was compared directly to **e**_**3**_^**DTI**^ and **v**_**3**_^**ST**^ for 200 μm DTI-ST-boxes which have simple laminar architecture.

## Results

### Cardiac *ex vivo* contractile state

In order to assess the cardiac contractile state of the fixed *ex vivo* hearts, dimensions were compared to cardiac dimensions predicted by indexing to body mass [37] in Table 3. The interventricular septum wall thickness and left ventricle posterior wall thickness are approximately twice the predicted systolic wall thicknesses. The left ventricle cavity diameter is 5% greater than the predicted systolic left ventricle cavity diameter.

**Table 3:** **Summary statistics quantifying** ***ex vivo*** **left ventricle wall thickness and left ventricle chamber diameter with comparison to predicted** ***in vivo*** **values from body mass**

**Ventricular wall thickness/chamber-diameter measured**	**Measured thickness in** ***ex vivo*** **FLASH**	**Predicted** ***in vivo*** **diastolic thickness**	**Predicted diastolic thickness as percentage of measured**	**Predicted** ***in vivo*** **systolic thickness**	**Predicted systolic thickness as percentage of measured**
**Interventricular septum**	3.0 ± 0.1	1.0 ± 0.0	33.3%	1.7 ± 0.0	56.7%
**Left ventricular posterior wall**	3.5 ± 0.3	1.3 ± 0.0	37.1%	1.8 ± 0.1	51.4%
**Left ventricular diameter**	3.7 ± 0.3	6.2 ± 0.1	168.6%	3.5 ± 0.0	94.6%

Data in this table allows comparison of the state of the fixed *ex vivo* hearts with *in vivo* contraction states (diastolic/systolic). The *in vivo* ventricular wall thicknesses are predicted from body-mass using the published equations (fitted to echocardiographic data) [37]. To assist comparison the predicted *in vivo* diastolic and systolic thicknesses are expressed as a percentage of the measured *ex vivo* FLASH thickness. The values listed for the thicknesses are mean ± SD. FLASH: fast low angle shot.

### Laminar structure revealed in FLASH

**Figure 4:**
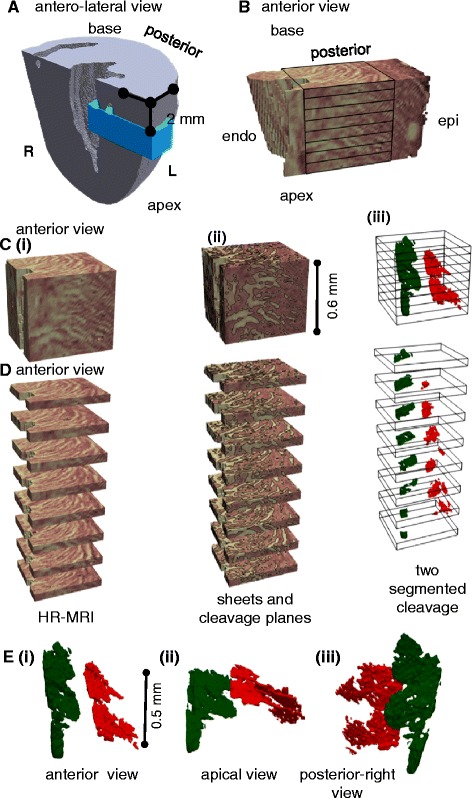
**FLASH image data. A** – the location of the lateral ROI is shown. **B** – visualization of the laminar architecture of a selected ROI in the lateral left ventricle wall. Myolaminae are colored in pink and sheetlet interstices in yellow. A cuboid divided into slices shows the region which will be explored in detail. **C** – the cuboid from (**B**) is shown **(i)** in isolation, **(ii)** with weighted shading to divide the image into laminae and sheetlet interstices, which are further separated by contour lines **(iii)** the tissue is rendered transparent, so that the structure of sheetlet interstices segmented within the ROI volume can be seen. **D** – the ROI from (**C**) is split (exploded) into slices to show the structure of the sheetlet interstices. **E** – the structure of the sheetlet interstices are shown in the same orientation as in the other images **(i)**, and in **(ii) & (iii)** rotated to reveal the branching structure of the sheetlet interstices. Image data from FLASH scan #8. L: left; R: right; endo: endocardium; epi: epicardium; ROI: region of interest; FLASH: fast low angle shot. The symbols for vectors and derived angles are defined in Table 2.

**Figure 5:**
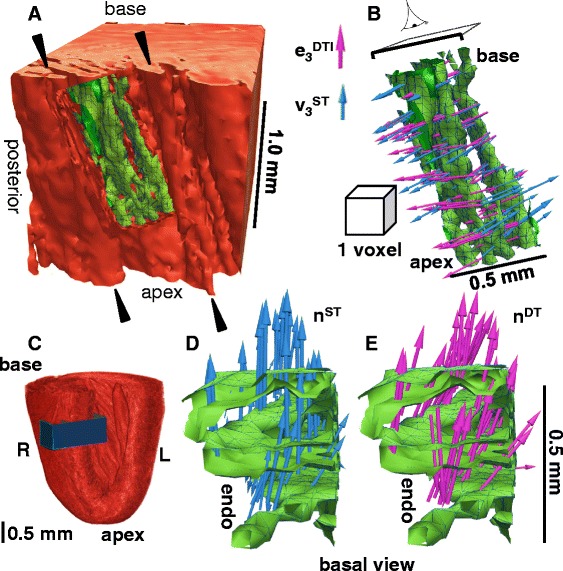
**Visualization of the FLASH laminar architecture in a left ventricle septal ROI. A** - The ROI is viewed so that the endocardial trabeculation is seen (black arrowheads); this trabeculation is continuous with the ventricular laminar architecture. In a small region of the myocardium the laminae have been segmented using an intensity threshold contour, and the 3D contour is colored green. **B** – the structure of these segmented contours are shown in the same orientation as in **(A)** and the ST (blue) and DTI (purple) putative sheetlet-normal vector orientations (**e**_**3**_^**DTI**^ and **v**_**3**_^**ST**^) are shown on the sheetlet interstices. The size of a DTI voxel (200 μm isotropic) is shown. **C** – the cardiac location of the septal ROI. **D** – a view from the cardiac base onto the segmented laminae is shown, with the ST sheetlet normal vectors (blue). **E** – likewise, with the DTI sheetlet normal vectors (purple). DTI: Scan #1, 6-direction, b = 1000 s/mm^2^; ST: Scan #8; DTW = 3, STW = 3. R: right; L: left; endo: endocardium; FLASH: fast low angle shot; ST: structure tensor of FLASH data; DTI: diffusion tensor magnetic resonance imaging; DTW: derivative template width STW: smoothing template width. The symbols for vectors and derived angles are defined in Table 2.

**Figure 6:**
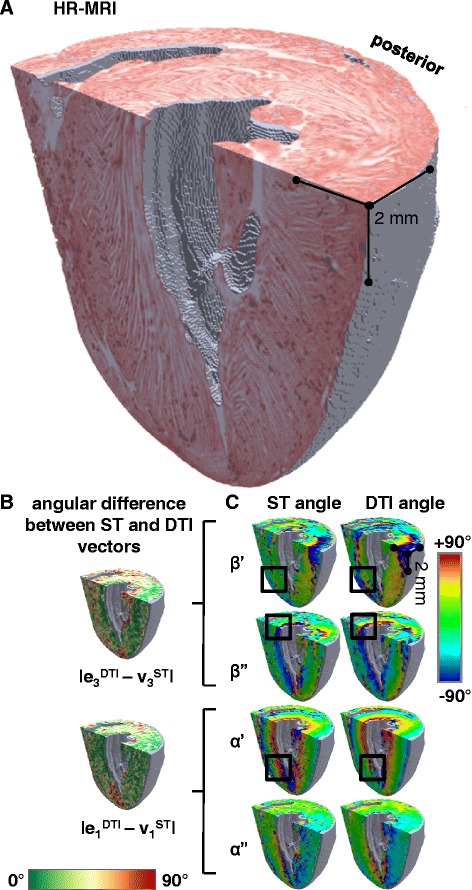
**Visualization of the 3D laminar architecture in FLASH, comparison between ST and DTI putative sheetlet-normal and myocyte orientation vectors and angles. A** – FLASH volume cropped to remove the heart base and the anterior ventricles. The sheetlets are colored red, and the sheetlet-interstices are white. The complex pattern of the laminae is seen, but the myocyte orientation cannot be directly observed. **B** - angle between the DTI (6-direction) and ST putative sheetlet-normal vectors and putative myocyte-orientation vectors, which are colored according to the 0° to +90° scale shown. **C** – the ST and DTI putative sheetlet-normal elevation (β’), sheetlet-normal transverse (β”), putative myocyte helix angle (α’), and putative myocyte transverse angle (α”), angle maps, which are colored according to the −90° to +90° scale shown. DTI: Scan #1, 6-direction, b = 1000 s/mm^2^; ST: Scan #8, DTW = 3, STW = 3. FLASH: fast low angle shot; ST: structure tensor of FLASH data; DTI: diffusion tensor magnetic resonance imaging; DTW: derivative template width STW: smoothing template width. The symbols for vectors and derived angles are defined in Table 2.

The FLASH of the laminar architecture is visualized in detail in Figures 3, 4, 5, 6 and in Additional file 2: Movie 1. We also visualize this structure in detail in a related previous study [11] which contains images and movies complementary to the views provided here. In contrast-enhanced FLASH images the myocardial cells exclude Gd-DTPA and appear dark (low-intensity), and the sheetlet-interstices contain Gd-DTPA and appear bright (high-intensity). To aid visualization, in Figure 4 the myocardial tissue is false colored red and the sheetlet interstices yellow. Figure 6A and Additional file 2: Movie 1 show that laminar structure is visible throughout much of the myocardium, and is particularly prominent in the sub-endocardium, and is less prominent in the sub-epicardium. A transmural cuboidal ROI in the lateral myocardial wall has been visualized in detail (Figure 4), and within this region the sheetlet-interstices architecture was segmented using the FI method. This approach is visualized from left to right across Figure 4C and D. When sheetlet-interstices are segmented in this manner the resultant regions of interconnected sheetlet interstices are plainly laminar, and have a complex interconnecting meshed structure with branches between adjacent laminar levels. In Figure 4C and D the visualized sheetlet-interstices have been limited in extension to a local region. It is, however, important to note that the visualized structures are integrated into the mesh of continuous myolaminar structure, i.e. the connected myolaminae and connected sheetlet interstices continue beyond the regions isolated here, as discussed in [11]. Two segmented sheetlet-interstices orientations are shown, with one in green (sub-endocardial) and one in red (sub-epicardial). In Figure 4D, the cuboid ROI has been virtually sliced (an exploded view) to show the structure of the laminae and sheetlet interstices through the volume, and the isolated sheetlet interstices are shown in Figure 4E, demonstrating that these sheetlet interstices do not have the same orientation with respect to the cardiac coordinate system. In the sub-epicardium two levels of parallel sheetlet interstices have been segmented together (shown in red) which are intimately and multiply joined, and cannot truly be considered a ‘single’ discrete sheetlet-interstice. In summary, this figure shows that contrast enhanced FLASH of the rat heart produces images of the orthotropic laminar structure of the myocardium, that the laminar architecture is absent (or not possible to define) in much of the sub-epicardium, and that this laminar architecture is highly branching. Elsewhere we have shown that the laminar architecture in FLASH corresponds to the laminar architecture in 2D histology images and that the overall laminar architecture is similar between rat hearts [11].

### Quantification myolaminar orientation in ST and DTI

In order to compare whole-heart myolaminar structure measured by ST/FLASH and by DTI the sheetlet-normal angles were visualized on the cardiac volume after long-axis and short–axis cropping of the full image, alongside the FLASH structure (Figure 6A-C; the corresponding sheetlet in-plane angles are visualized in Additional file 3: Figure DS1). To allow direct comparison eigenanalysis was applied to the ST data at the same resolution as the DTI data (64 × 64 × 128 tensors). Additional file 2: Movie 1 and Additional file 4: Movie 2 show animated longitudinal slices of the corresponding FLASH structure and derived angles from a second rat heart. Colored images of sheetlet orientation are widely used in the cardiac structure literature [8,20,32,34,38], and show quantitative information but are challenging to interpret. The images provide limited information about 3D structural complexity and provide no information about the connectivity of spatial scales of laminae. Therefore laminar structure has been directly visualized in a septal transmural ROI, and this is shown together with the ST/FLASH **v**_**3**_^**ST**^ and DTI **e**_**3**_^**DTI**^**,** both putative measures of **n,** in Figure 5.

### Comparison of ST and DTI laminar orientation to FI

**Figure 7:**
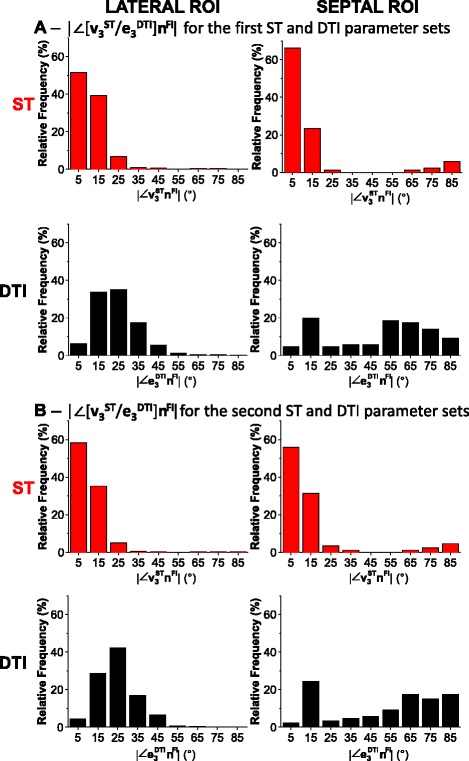
**ST and DTI putative sheetlet normal orientations compared voxel-wise to the FI normal** **(|∠ [v**_**3**_^**ST**^ **/e**_**3**_^**DTI**^ **]n** ^**FI**^ **|).** These frequency distributions summarize the orientations from the interactively segmented sheetlets of Figure 3 for the lateral ROI (left) and for the septal ROI (right). **A** - distributions produced for the first set of imaging/image-processing parameters compared to FI (DTI: Scan #1, 6-direction, b = 1000 s/mm^2^; ST: Scan #8, DTW = 3, STW = 3). **B** - distributions for the second set of imaging/image-processing parameters compared to FI (DTI: Scan #2, 12-direction, b = 1000 s/mm^2^; ST: Scan #8, DTW = 5, STW = 5) allow assessment of sensitivity of the measured laminar normals to these parameters. Note: the deviation angles shown in these histograms are not on a circular scale as they are absolute values and 0° ≠ 90°. FLASH: fast low angle shot; ST: structure tensor of FLASH data; DTI: diffusion tensor magnetic resonance imaging; FI: FLASH isosurface data; DTW: derivative template width STW: smoothing template width. The symbols for vectors and derived angles are defined in Table 2.

We compared ST and DTI laminar orientation measurement respectively against a direct interactive visualization approach (FI). It can be seen in the example voxel in Figure 3F that the laminar structure has a clearly defined simple orientation, and that **v**_**3**_^**ST**^ is much closer than **e**_**3**_^**DTI**^ to **n**^**FI**^ (|∠**v**_**3**_^**ST**^**n**^**FI**^| = 7.8°; |∠**e**_**3**_^**DTI**^**n**^**FI**^| = 19.6°). This individual voxel comparison is for the purpose of illustrating the approach, and no conclusions can be drawn from this voxel alone. However, the same approach is visualized qualitatively in Figure 5, and then applied to quantify |∠**v**_**3**_^**ST**^**n**^**FI**^| and |∠**e**_**3**_^**DT**^**n**^**FI**^| for the whole of the lateral and septal ROIs (Figure 7A).

In Figure 7A and Table 4 comparisons of ST and DTI derived sheetlet normal orientations to the FI normal are shown from the starting ST and DTI parameters sets (DTI: Scan #1, 6-direction, b = 1000 s/mm^2^ ; ST: Scan #8, DTW = 3, STW = 3). The angle reported is the absolute angle of deviation between **v**_**3**_^**ST**^ or **e**_**3**_^**DTI**^ and **n**^**FI**^, here expressed in the short-hand notation |∠[**v**_**3**_^**ST**^/**e**_**3**_^**DT**^]**n**^**FI**^|, and defined as |cos^−1^ [**v**_**3**_^**ST**^/**e**_**3**_^**DTI**^].**n**^**FI**^)|. By definition 0° ≤ | [**v**_**3**_^**ST**^/**e**_**3**_^**DTI**^].**n**^**FI**^| ≤ 90°. In the lateral ROI, the distribution of the ST vector difference |∠**v**_**3**_^**ST**^**n**^**FI**^| is narrow, unimodal and centered close to 0°, while the DTI vector difference |∠**e**_**3**_^**DTI**^**n**^**FI**^| is broader, unimodal and centered on 22.5°. In the septal ROI, the ST vector difference |∠**v**_**3**_^**ST**^**n**^**FI**^| is bimodal with a narrow dominant mode centered near 0° (90.7% of voxels) and a small second mode centered near 90° (9.3% of voxels). The DTI vector difference |∠**e**_**3**_^**DTI**^**n**^**FI**^| is also bimodal but with a broad dominant mode centered near 65° (65.1% of voxels) and a small second narrow mode centered on 15° (29.1% of voxels). This data is summarized in Additional file 5: Figure DS5 (Additional file 1: Supplemental Results) where summary data is also shown for the anterior and posterior ROI.

**Table 4:** **Summary statistics of voxel-wise comparison of ST/DTI sheetlet normal to FI**

**#** ^**a**^	**STW/DTW**	**CMR**	**n-dir**	**b-value (s/mm** ^**2**^ **)**	**|∠[v**_**3**_^**ST**^ ** or e**_**3**_^**DTI**^ ** ]n** ^**FI**^ **| median ± I.Q.R. (°)**
**LATERAL ROI**					
First ST and DTI parameter sets					
**8**	3/3	T1W	NA	NA	8.7 ± 7.8
**1**	NA	DTI	6	1000	22.5 ± 13.8
Second ST and DTI parameter sets					
**8**	5/5	T1W	NA	NA	9.1 ± 7.4
**2**	NA	DTI	12	1000	24.1 ± 11.9
**SEPTAL ROI**					
First ST and DTI parameter sets					
**8**	3/3	T1W	NA	NA	7.4 ± 8.7
**1**	NA	DTI	6	1000	54.4 ± 47.3
Second ST and DTI parameter sets					
**8**	5/5	T1W	NA	NA	8.5 ± 9.7
**2**	NA	DTI	12	1000	60.5 ± 53.4

These statistics relate to the histograms in Figure 7 of the voxel-wise comparison of ST and DTI determined sheetlet putative normal orientations to the FI normal for the lateral ROI (left) and for the septal ROI (right). ; ^a^ – scan # as defined in Table 1; NA: Not Applicable; FLASH: fast low angle shot; ST: structure tensor of FLASH data; DTI: diffusion tensor magnetic resonance imaging; T1W is T1-weighted FLASH; n-dir: number of diffusion directions; ROI: region(s) of interest. FI: FLASH isosurface data; DTW: derivative template width STW: smoothing template width. The symbols for vectors and derived angles are defined in Table 2.

### Quantification of the confidence in the sorting of laminar eigenvectors

**Figure 8:**
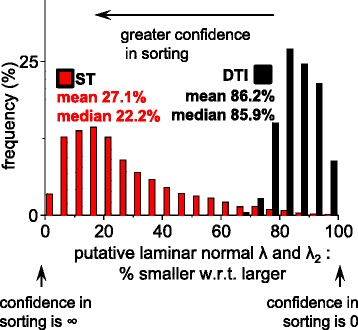
**Exploration of the relative magnitudes of the laminar eigenvalues in the lateral ROI.** In order to assess for DTI and for ST whether meaningful sorting of the putative laminar normal eigenvector from the intermediate-eigenvector is possible the magnitudes of the putative laminar normal eigenvalue was compared to the λ_2_ (i.e. for ST λ_1_ was compared to λ_2_ and for DTI λ_3,_ was compared to λ_2_). In each case the smaller eigenvalue is expressed as a percentage of the larger eigenvalue, where 100% indicates identity, and that there is no confidence in sorting the putative laminar normal orientation from the intermediate-eigenvector orientation, and approaching 0% the confidence in sorting is high. DTI: Scan #2, 12-direction, b = 1000 s/mm^2^; ST: Scan #8, DTW = 3, STW = 3). Data in this figure is from the lateral ROI which was visualized and compared to FI laminar orientations in Figure 3&7. FLASH: fast low angle shot; ST: structure tensor of FLASH data; DTI: diffusion tensor magnetic resonance imaging; ROI: region of interest; DTW: derivative template width STW: smoothing template width. The symbols for vectors and derived angles are defined in Table 2.

Eigenvector misassignment (missorting) is the assignment of an eigenvector to the incorrect structural feature due to imaging noise and small differences in eigenvalue magnitudes. In order to explore whether ST or DTI eigenvector misassignment was a source of error in myolaminar measurement the distributions of eigenvalue ratios from the lateral ROI were examined (Figure 8). Distributions of the ratios of values are plotted rather than raw values so as to preserve the relationship between eigenvalue pairs. In Figure 8, in the lateral ROI, there was little difference between the DTI sheetlet and sheetlet normal eigenvalues (9% of voxels have less than 5% difference in λ_2_ and λ_3_; a further 21% of voxels have less than 10% difference between λ_2_ and λ_3_; i.e. in 30% of voxels λ_3_ is at least 85% of λ_2_). The DTI median (±IQR) and mean (±SD) difference between laminar eigenvalues (the laminar eigenvalues are for λ_2_ and λ_3_ for DTI and λ_1_ and λ_2_ for ST) are 14.1% ± 9.8% and 13.8 ± 6.0% respectively. This corresponds to a median difference of 77.8% ± 23.9% and a mean difference of 72.8% ± 18.7% for ST. The sets of eigenvalues which correspond to the myolaminae are not the same for DTI and ST (for DTI: λ_2_ and λ_3_, for ST: λ_1_ and λ_2_). An implication of this much greater separation of ST laminar eigenvalues than DTI laminar eigenvalues is that misclassifications of **e**_**3**_^**DTI**^ and **e**_**2**_^**DTI**^ are more likely than misclassifications of **v**_**3**_^**ST**^ and **v**_**2**_^**ST**^.

### Direct comparison of ST and DTI laminar orientation

**Figure 9:**
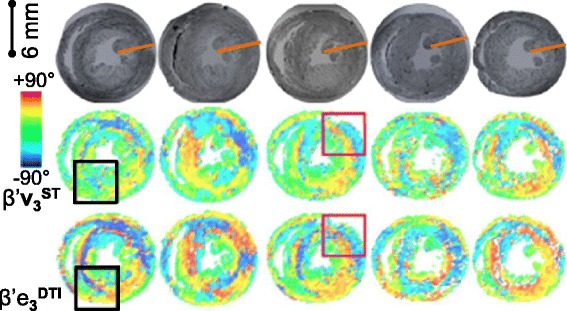
**The ST and DTI putative sheetlet-normal angles are compared for 5 rat hearts.** The **v**_**3**_^**ST**^ and **e**_**3**_^**DTI**^ elevation (β’) angle maps of an equatorial short-axis slice are colored according to the −90° to +90° scale. Regions of similar and differing laminar normal orientation are shown in the magenta and black boxes respectively. The transmural orange line on the FLASH images indicates the transmural span quantified in Figure 14. DTI: Scan #1, 6-direction, b = 1000 s/mm^2^; ST: Scan #8, DTW = 3, STW = 3. FLASH: fast low angle shot; ST: structure tensor of FLASH data; DTI: diffusion tensor magnetic resonance imaging; DTW: derivative template width STW: smoothing template width. The symbols for vectors and derived angles are defined in Table 2. The associated angle maps for the **v**_**3**_^**ST**^ and **e**_**3**_^**DTI**^ transverse (β”) angle are in Additional file 6: Figure DS2.

The comparison of **v**_**3**_^**ST**^ and **e**_**3**_^**DTI**^ to **n**^**FI**^ is limited as the FI method requires interactive segmentation and visualization and was carried out on four equatorial ROI of one rat heart. The analysis was extended to a series of 5 hearts from age matched rats in Figure 9, by direct comparison of DTI and ST laminar orientations.

**Figure 10:**
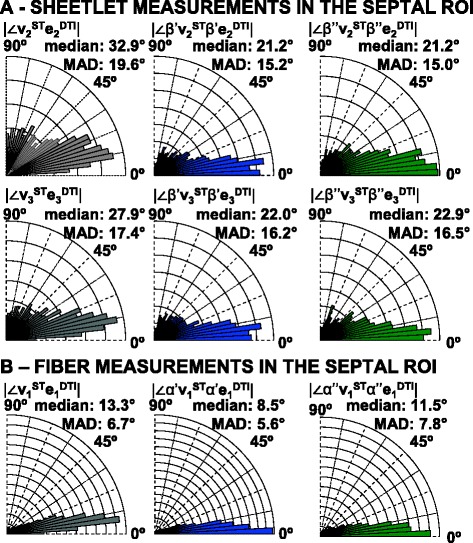
**Equatorial septal ROI distributions of angle differences between the ST and DTI vectors/orientation angles. A** – deviation angles |∠**v**_**3**_^**ST**^ **e**_**3**_^**DTI**^| and |∠**v**_**2**_^**ST**^ **e**_**2**_^**DTI**^| are shown, alongside the corresponding distributions of |∠β’**v**_**3**_^**ST**^β’**e**_**3**_^**DTI**^|, |∠β”**v**_**3**_^**ST**^β”**e**_**3**_^**DTI**^|, |∠β’**v**_**2**_^**ST**^β’**e**_**2**_^**DTI**^|, |∠β”**v**_**2**_^**ST**^β”**e**_**2**_^**DTI**^|. **B** – deviation angles |∠**v**_**1**_^**ST**^ **e**_**1**_^**DTI**^| and |∠**v**_**1**_^**ST**^ **e**_**1**_^**DTI**^| are shown, alongside the corresponding distributions of |∠α’**v**_**1**_^**ST**^α’**e**_**1**_^**DTI**^|, |∠α”**v**_**1**_^**ST**^α”**e**_**1**_^**DTI**^|. DTI: Scan #1, 6-direction, b = 1000 s/mm^2^; ST: Scan #8; DTW = 3; STW = 3. FLASH: fast low angle shot; ST: structure tensor of FLASH data; DTI: diffusion tensor magnetic resonance imaging; DTW: derivative template width STW: smoothing template width; MAD: median absolute deviation; ROI: region(s) of interest. The symbols for vectors and derived angles are defined in Table 2. The corresponding distributions for the equatorial lateral ROI are in Additional file 7: Figure DS3.

In Figure 9 the putative laminar normal β’ angles are colored (on the −90° to +90° color scale) for an equatorial short-axis slice from 5 rat hearts, after whole-heart registration. The corresponding FLASH images of laminar architecture are shown for comparison. The equivalent visualizations of β” angles are in Additional file 6: Figure DS2 (see Additional file 1: Supplemental Results). As for the 3D visualization in Figure 6, it is clear from inspection that there are similarities between the sheetlet orientation maps from DTI and from ST, but also areas of difference. To quantitatively investigate these observed differences between ST and DTI myolaminar orientation, the absolute differences between the putative sheetlet-normal eigenvectors and derived orientations (**v**_**3**_^**ST**^, β’**v**_**3**_^**ST**^, β”**v**_**3**_^**ST**^; and **e**_**3**_^**DTI**^, β’**e**_**3**_^**DTI**^, β”**e**_**3**_^**DTI**^) and the sheetlet in-plane vectors and angles are explored in rose diagrams for the septal ROI from one heart in Figure 10A. The properties of the sheetlet in-plane vectors and angles are discussed in the results supplement. As the myolaminar vectors are axial (their orientation is in the range of 180°) the angle difference between them is in the range 0 - 90°. Therefore, the angle difference plots are quadrant rose diagrams. These statistical diagrams have the following key characteristics: (i) for near-identical distributions they are narrow and centered at 0°; (ii) measures with large systematic error exhibit narrow distributions that are not centered at 0°; and, (iii) for randomly associated vectors the distributions are evenly spread across the 0 - 90° range of the quadrant. The center is represented by the median, and the spread by the median absolute deviation (MAD). Associated rose diagrams for the lateral ROI are in the Additional file 7: Figure DS3 (Additional file 1). |∠**v**_**3**_^**ST**^**e**_**3**_^**DTI**^| has a spread out distribution skewed towards lower values, (median ± MAD: 27.9 ± 17.4°). The same pattern is observed in the derived sheetlet-normal elevation angles |∠β’**v**_**3**_^**ST**^β’**e**_**3**_^**DTI**^|, but the differences are less marked. It can be seen that the general properties of the angle difference of the vector are also observed in the distributions of the elevation and transverse angles. The patterns described for the septal ROI are also observed in the lateral ROI (Additional file 7: Figure DS3A), but to a lesser degree. The local myocyte-orientation vector comparisons in Figure 10B and Additional file 7: Figure DS3B are discussed later in the manuscript.

**Figure 11:**
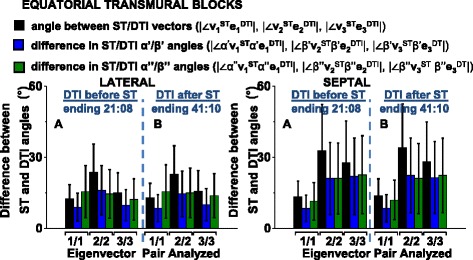
**Differences between ST and DTI vary depending on cardiac location and are stable over time.** Results are presented by region (lateral, septal) showing the deviation between the ST and the corresponding DTI eigenvector orientations pairs (of **v**_**1**_^**ST**^ **e**_**1**_^**DTI**^, **v**_**2**_^**ST**^ **e**_**2**_^**DTI**^ and **v**_**3**_^**ST**^ **e**_**3**_^**DTI**^ ), and the difference between the associated vector elevation and transverse angles. Side **A** (left) of each histogram are angles from comparison of ST to a DTI image taken in the 2 hours BEFORE the FLASH (Scan #7). Side **B** are from comparison of ST to a DTI image taken in the 2 hours AFTER the FLASH (Scan #9). DTI: 6-direction, b = 1000 s/mm^2^; ST: Scan #8, DTW = 3, STW = 3. FLASH: fast low angle shot; ST: structure tensor of FLASH data; DTI: diffusion tensor magnetic resonance imaging; DTW: derivative template width STW: smoothing template width. The symbols for vectors and derived angles are defined in Table 2. The corresponding distributions for the posterior and anterior ROI are in Additional file 8: Figure DS4.

In Figure 11 the data in the quadrant rose plots of the lateral and septal ROI is combined into a histogram of deviation angle mean ± SD for each of the: angle between **v**_**2**_^**ST**^ and **e**_**2**_^**DTI**^ (i); the putative sheetlet in-plane vector elevation angle |∠β’**v**_**2**_^**ST**^β’**e**_**2**_^**DTI**^| (ii) and transverse angle |∠β”**v**_**2**_^**ST**^β”**e**_**2**_^**DTI**^| (iii); angle between **v**_**3**_^**ST**^ and **e**_**3**_^**DTI**^ (iv); the putative sheetlet-normal vector elevation angle |∠β’**v**_**3**_^**ST**^β’**e**_**3**_^**DTI**^| (v) and, transverse angle |∠β”**v**_**3**_^**ST**^β”**e**_**3**_^**DTI**^| (vi). Corresponding data for the posterior and anterior ROI are shown in Additional file 8: Figure DS4. Data is also shown in Figure 11 for **v**_**1**_^**ST**^ and **e**_**1**_^**DTI**^ and the angles of local myocyte orientation, and this is discussed in the section ‘*Direct comparison of ST and DTI local myocyte orientation’* below. The stability of the measurements over time is discussed in the Digital Supplement (section ‘*DTI and ST sensitivity analysis*’). The general patterns described for the septal ROI in Figure 10 of high bias and variation (bias greater than 20°, SD greater than 20°) of **e**_**3**_^**DTI**^ with respect to **v**_**3**_^**ST**^ are also observed, but to a lesser degree, in the lateral, anterior and posterior ROI (Figure 11 and Additional file 8: Figure DS4). There are no absolute cut-off values for comparing deviation angle bias or variation. The degree of similarity between the DTI and ST laminar and laminar normal orientations varies depending on the ROI studied. The lowest value of |∠**v**_**3**_^**ST**^**e**_**3**_^**DTI**^| (i.e. the best agreement between ST and DTI) is 15 ± 10° (in the lateral ROI) and the maximum value of |∠**v**_**3**_^**ST**^**e**_**3**_^**DTI**^| is 30 ± 15°. This analysis (unlike the comparison to **n**^**FI**^ values above) includes all ST and DTI 200 μm voxels without filtering based on the simplicity/complexity of the contained laminar structure.

### Direct comparison of ST and DTI local myocyte orientation

**Figure 12:**
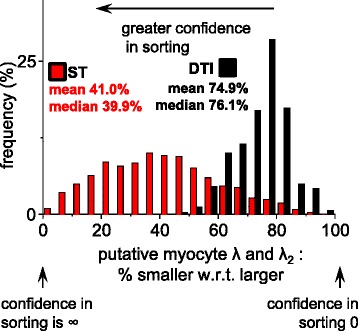
**Exploration of the relative magnitudes of the putative myocyte orientation eigenvalues in the lateral ROI.** In order to assess for DTI and for ST whether meaningful sorting of the putative myocyte eigenvector from the intermediate-eigenvector is possible the magnitudes of the putative myocyte orientation eigenvalue was compared to the λ_2_ (i.e. for ST λ_3_ was compared to λ_2_ and for DTI λ_1,_ was compared to λ_2_). In each case the smaller eigenvalue was expressed as a percentage of the larger eigenvalue. 100% indicates identity and that there is no confidence in sorting the putative myocyte orientation from the intermediate-eigenvector orientation, and approaching 0% the confidence in sorting is high. DTI: Scan #2, 12-direction, b = 1000 s/mm^2^; ST: Scan #8, DTW = 3, STW = 3. Data in this figure is from the lateral ROI which was visualized and compared to FI laminar orientations in Figures 3 and 7. FLASH: fast low angle shot; ST: structure tensor of FLASH data; DTI: diffusion tensor magnetic resonance imaging; DTW: derivative template width STW: smoothing template width; ROI: region(s) of interest. The symbols for vectors and derived angles are defined in Table 2.

Unlike the case of myolaminar orientation, there is no method to directly determine local myocyte orientation from the FLASH data against which **e**_**1**_^**DTI**^ and **v**_**1**_^**ST**^ can be compared. This is because it is not possible for FLASH to resolve individual cardiac myocytes at 50 × 50 × 50 μm^3^ resolution. Therefore the putative myocyte orientation vectors **e**_**1**_^**DTI**^ and **v**_**1**_^**ST**^ are compared to each other. The first part of this comparison is to evaluate the basis for eigenvector assignment (in the same manner as was carried out for laminar eigenvectors in section ‘*Quantification of the confidence in the sorting of laminar eigenvectors*’ above. The relative magnitudes of the putative local myocyte eigenvalue are compared to the next closest eigenvalue (λ_2_) in Figure 12. It might be expected that there would be a stronger basis for sorting of **e**_**1**_^**DTI**^ from the other DTI eigenvectors than in sorting **v**_**1**_^**ST**^ from the other ST eigenvectors, as **e**_**1**_^**DTI**^ is a primary eigenvector. This is not the case as there is greater difference between λ_2_^ST^ and λ_3_^ST^ than between λ_2_^DTI^ and λ_1_^DTI^. The median difference between λ_2_^ST^ and λ_3_^ST^ is 61.1% ± 28.1%. This compares to a median difference of 23.9% ± 11.5%. As discussed above, the sets of eigenvalues which are relevant to assignment of the local myocyte orientation are not the same for DTI and ST (for DTI: λ_1_ and λ_2_, for ST: λ_2_ and λ_3_). An implication of this greater separation of the eigenvalues relevant to **v**_**1**_^**ST**^ assignment (λ_2_^ST^ and λ_3_^ST^) than the eigenvalues relevant to **e**_**1**_^**DTI**^ assignment (λ_2_^DTI^ and λ_1_^DTI^), is that misclassification of **e**_**1**_^**DTI**^ and **e**_**2**_^**DTI**^ is more probable than misclassification of **v**_**1**_^**ST**^ and **v**_**2**_^**ST**^. There is no absolute cut-off for the median difference between eigenvalues which is acceptable to allow confidence in the DTI or ST assignment of the putative local myocyte orientation (**m**). The order of the eigenvalue sets from greatest difference to least difference is: (i) λ_2_^ST^ with λ_1_^ST^; (ii) λ_3_^ST^ with λ_2_^ST^; (iii) λ_2_^DTI^ with λ_1_^DTI^; and, (iv) λ_2_^DTI^ with λ_3_^DTI^.

**Figure 13:**
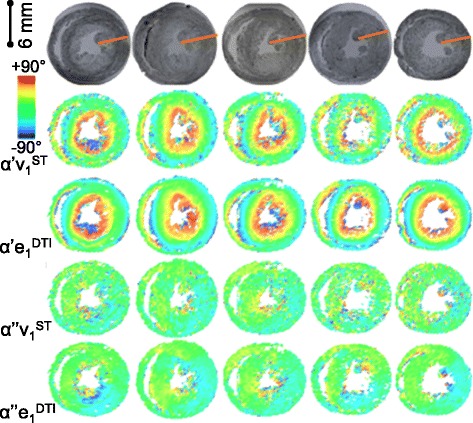
**The putative ST and DTI myocyte angles are compared for 5 rat hearts.** The putative myocyte helix angle α’ and transverse angle α” in an equatorial short-axis slice are colored according to the −90° to +90° scale shown. The transmural orange line on the FLASH images indicates the transmural span quantified in in Figure 14. DTI: Scan #1, 6-direction, b = 1000 s/mm^2^; ST: Scan #8, DTW = 3, STW = 3. FLASH: fast low angle shot; ST: structure tensor of FLASH data; DTI: diffusion tensor magnetic resonance imaging; DTW: derivative template width STW: smoothing template width. The symbols for vectors and derived angles are defined in Table 2.

The ST and DTI local myocyte angles were compared in 3D visualizations alongside the images of the FLASH images in Figure 6C and for a second heart in Additional file 9: Movie 3. The DTI local myocyte helix-angle (α’**e**_**1**_^**DTI**^) follows the familiar smooth transmural change in orientation from large positive angles at the endocardium (+65° ± 5°) to negative angles at the epicardium (−65° ± 20°) [8]. The smooth change is observed around the circumference of the short-axis slice and in the long-axis views from base to apex. There is greater noise in the ST than the DTI, both for α’ and α”. In Figure 13 the similarity between ST and DTI for both α’ and α” is across the entire short-axis slice. In Figure 6 this similarity between ST and DTI α’ and α” is across most of the long-axis view, however, there are also small regions of difference in α^’^ in the septal-apex, as identified with the black square in Figure 6C. This 3D qualitative analysis in one heart is extended to 2D qualitative analysis in five registered hearts in Figure 13. In Figure 13 α’**e**_**1**_^**DTI**^ and α”**e**_**1**_^**DTI**^ are visualized in the same equatorial short-axis slice for 5 hearts showing that: (i) α’**e**_**1**_^**DTI**^ and α’**v**_**1**_^**ST**^ are similar within individual rats; (ii) α”**e**_**1**_^**DTI**^ and α”**v**_**1**_^**ST**^ are similar within individual rats; (iii) α’ and α” are consistent between rat hearts, whether measured by ST (**v**_**1**_^**ST**^) or DTI (**e**_**1**_^**DTI**^); (iv) both α’**e**_**1**_^**DTI**^ and α”**e**_**1**_^**DTI**^ are very similar to rat α’**e**_**1**_^**DTI**^ and α”**e**_**1**_^**DTI**^ reported in the literature [39]. As observed in the single heart volumetric analysis (above, Figure 6C) the ST measured local myocyte angles are globally similar to the DTI local myocyte angles, and likewise, there is greater noise in the ST data than in the DTI data. There are no regions of large difference between α’**v**_**1**_^**ST**^ to α’**e**_**1**_^**DTI**^ in the equatorial slices (the identified region of difference in α’ in Figure 6C was limited to the apex).

**Figure 14:**
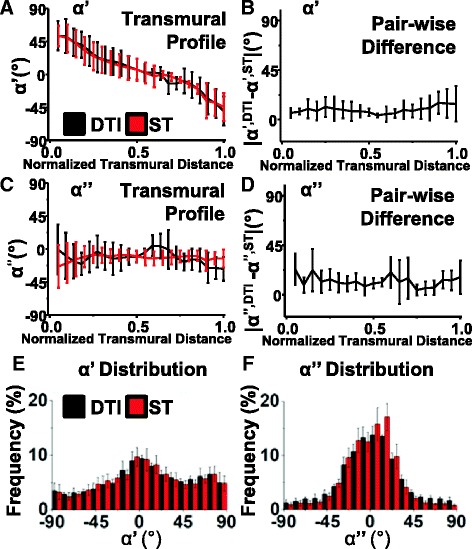
**Quantitative comparison of ST and DTI putative myocyte angles of the 5 rat hearts. A -** The transmural profiles of the putative myocyte helix angle (α’). **B -** The transmural pair-wise difference plots for the putative myocyte helix angle |α’**e**_**1**_^**DTI**^ - α’**v**_**1**_^**ST**^|. **C -** The transmural profiles for the putative myocyte transverse angle (α”). **D -** The transmural pair-wise difference plots for the putative myocyte transverse angle |α”**e**_**1**_^**DTI**^ – α”**v**_**1**_^**ST**^|. **E -** The distribution of the putative myocyte helix angles (α’). **F -** The distribution of the putative myocyte transverse angles (α”). DTI: Scan #1, 6-direction, b = 1000 s/mm^2^; ST: Scan #8, DTW = 3, STW = 3. FLASH: fast low angle shot; ST: structure tensor of FLASH data; DTI: diffusion tensor magnetic resonance imaging; DTW: derivative template width STW: smoothing template width. The symbols for vectors and derived angles are defined in Table 2.

The qualitative comparison of local myocyte orientation in five hearts in Figure 13 is quantified in Figure 14A for the same lateral transmural equatorial region of the same 5 rat hearts. Transmural profiles are commonly used to present local myocyte angles and the rationale for their use is that the local myocyte angles, unlike sheetlet angles, are largely a function of the transmural position (i.e. can be approximated from a simple equation, with the only variable being the transmural distance [13]. In Figure 14 the transmural profile of the α’ from both **e**_**1**_^**DTI**^ and **v**_**1**_^**STI**^ follows the classically described pattern [13] and the pattern previously described for DTI [39] and the profiles for α’**e**_**1**_^**DTI**^ and α’**v**_**1**_^**ST**^ are remarkably similar, as are the profiles for α”**e**_**1**_^DTI^ and α”**v**_**1**_^**ST**^. Greater angular standard deviations for ST **v**_**1**_ than for DTI **e**_**1**_ for both α^’^ and α^”^ are a consequence of the greater noise apparent in the slice images in Figure 13. An alternative comparative measure is used in Figure 14B where the transmural profile of the α’ pair-wise difference is plotted. This is the transmural profile of the mean and SD of |α’**v**_**1**_^**ST**^ – α’**e**_**1**_^**DTI**^| calculated for each voxel. This measure is particularly appropriate for this study where DTI and ST describe precisely the same voxel matrices. Perfectly matched measures will have a profile parallel to the x-axis at 0°. The pair-wise difference in α’ is small in the sub-endocardium and mid-myocardium: 5-10° ± 15°, increasing in the sub-epicardium: 15° ± 15° in the sub-epicardium. In Figure 14C the α” transmural profile is close to 0° and the α” pair-wise difference transmural profile is close to 5° ± 10° (Figure 14D). The distributions of α’ and α” are shown in Figure 14E and F where there are very similar distributions of α’**v**_**1**_^**ST**^ (5.3° ± 3.6°) and α’**e**_**1**_^**DTI**^ (5.2° ± 2.7°) and of α”**v**_**1**_^**ST**^ (3.1 ± 7.0) and α”**e**_**1**_^**DTI**^ (5.7 ± 9.0). The distribution of α’ is spread across the −90° to +90° range, with a higher frequency of angles close to 0° for both ST and DTI. The distribution of α” is unimodal, centered close to 0° and predominantly in the range −22.5° to +22.5°.

The comparative deviation angles of distributions of **v**_**1**_^**ST**^**: e**_**1**_^**DTI**^; α’**v**_**1**_^**ST**^: α’**e**_**1**_^**DTI**^ and α”**v**_**1**_^**ST**^: α”**e**_**1**_^**DTI**^ are explored in quadrant rose diagrams in Figure 10B (for the septal ROI) and in Additional file 7: Figure DS3B (for the lateral ROI). The distributions for |∠**v**_**1**_^**ST**^**e**_**1**_^**DTI**^| are unimodal and narrow in the septal ROI (median ± MAD**:** 13.3° ± 6.7°) and in the lateral ROI (median ± MAD**:** 12.6° ± 5.9°). The distributions of |∠α’**v**_**1**_^**ST**^:α’**e**_**1**_^**DTI**^| have the same form, being unimodal and narrow in the septal ROI (median ± MAD: 8.5° ± 5.6°) and in the lateral ROI (median ± MAD: 9.1° ± 5.8°). Likewise, the distributions of |∠α”**v**_**1**_^**ST**^:α”**e**_**1**_^**DTI**^| also have the same form, being unimodal and narrow in the septal ROI (median ± MAD**:** 11.5° ± 7.8°) and in the lateral ROI (median ± MAD**:** 15.6° ± 11.1°). In the septal ROI the distributions of |∠**v**_**1**_^**ST**^**e**_**1**_^**DTI**^| (the local myocyte orientation angles) are therefore in contrast to the distributions of |∠**v**_**2**_^**ST**^**e**_**2**_^**DTI**^| and |∠**v**_**3**_^**ST**^**e**_**3**_^**DTI**^| (and the associated sheetlet and sheetlet normal angles), the latter having greater bias and variation. This pattern is also seen in the lateral ROI in Additional file 7: Figure DS3 and in the anterior and posterior ROI (Additional file 8: Figure DS4), but to a lesser degree.

### DTI and ST sensitivity analysis

A series of imaging experiments carried out to explore sensitivity of ST and DTI to imaging parameters are presented in the Digital Supplement (section *DTI and ST sensitivity analysis*) and in Additional file 10: Figure DS6. We showed that the overall DTI sensitivity to time post fixation is low; to b-value is moderate (with b-value scaled by change in diffusion gradient amplitude with fixed diffusion gradient separation time of Δ = 11.5 ms); and to number of diffusion directions is low. Optimal DTI imaging parameters were b = 1000 mm/s^2^ and 12 diffusion directions with post-fixation time (up to 72 hours) not being an important factor. ST was not sensitive to image processing parameters in the range explored.

## Discussion

This study compares DTI myolaminar measurement against direct measurement of myolaminar orientation from FLASH of the fixed rat heart. The Digital Supplement (Additional file 1) has further discussion in the sections *Discussion of the Results of Other Validation Studies* and *Eigenvalue Comparison*.

### The benefits of validating against FLASH

This approach of measuring DTI myolaminar orientation performance and sensitivity referenced to direct measurement in FLASH (the FI method) has several advantages over previously adopted methods. This is discussed in more detail in the Digital Supplement (section *Discussion of the results of other validation studies*). Our approach has the benefit that: (i) direct comparison of DTI to FI method assumes no cardiac model of local myocyte orientation or of the relationship between local myocyte orientation and myolaminar orientation; and, (ii) no registration is required as FLASH imaging and DTI imaging are carried out sequentially without moving the heart, and using coincident CMR imaging matrices. Unlike all previous methods of validating DTI myolaminar measurements the method we use does not rely on first estimating **n** through prior knowledge about **m**. We directly measure the 3D orientation of the laminar normal from images (**n**^**FI**^) and compare this directly to the putative measure of sheetlet normal orientation **e**_**3**_^**DTI**^. The rationale for this approach is firstly that it is simple and secondly that it follows directly from the initial description of sheetlets (i.e. from examining images). This simple approach was possible as the 3D myolaminar structure is directly visible and well-defined in the contrast-enhanced FLASH [11] which is in the same imaging frame as the DTI. Cardiac laminar structure was first described from histological observations, and from using 3D reconstructive methods to show that there was local branching sheetlet structure, which extended in three-dimensions and was divided by sheetlet-interstices, which likewise extended in 3D as a branching network. In this study the sheetlets are defined as the clearly visible local stacked branching structures of low signal intensity in FLASH and correspondingly the sheetlet-interstices are defined as the intermeshed local stacked branching structures of high FLASH signal intensity.

Previous validation studies have either used a 2D histological method followed by DTI [21] or DTI followed by a 2D histological method [40]. There are two important limitations in the use of 2D imaging for measuring myolaminar orientation. Due to the limited 2D view of the tissue, and due to sectioning artefact resulting in some cellular separation, it is possible to misinterpret the grain of the local myocyte direction as sheetlet interstices, and hence to measure spurious myolaminae/sheetlet-interstices orientations which have no correspondence to true myolaminae in the native heart (discussed in [40], [21]). Secondly, the orientation of myolaminae/sheetlet interstices cannot be directly measured in 2D images, only the intersection angle of the myolaminae/sheetlet-interstices with the section, as discussed in the section (Results of other validation studies) in the Digital Supplement. This seems counterintuitive, as the sheetlet-interstice grain on a 2D section results from the sheetlet-plane. However it is 2D cut through a 3D plane, and by definition it cannot directly give the orientation of the myolaminar plane. The measurement is a line of intersection of the cut section plane, and as such is a non-standard sheetlet angle. A standard sheetlet angle can be obtained by careful alignment of the section plane to a standard cardiac plane, and this allows either β’**e**_**2**_^**DTI**^ or β”**e**_**2**_^**DTI**^ to be measured but not both. A single image of the cut surface of the myocardium gives very limited information on the orientation of the myolaminae below.

Indirect strategies have been developed in order to measure laminar orientation from 2D sections in spite of these two important limitations. The first strategy is to use prior knowledge of local myocyte orientation, for example literature based descriptions or mathematical “rule-based“ models of the local cardiac local myocyte orientation [13] (rule-based models are myocyte helix and transverse angles determined by simple mathematical functions using the cardiac location as a parameter). The second strategy is to use prior knowledge of cardiac local myocyte/laminar association (the orthotropic model of cardiac structure) in order to reconstruct the sheetlet normal orientation. Both of these strategies are based upon good models of cardiac structure, but these are macroscopic models and are hence approximations of local structure, and their accuracy will vary depending on cardiac location. As such they are not a good method against which to assess DTI.

As FLASH/FI resolves sheetlets and sheetlet-interstices in 3D throughout the myocardium it is an objective basis for comparison of both **e**_**3**_^**DTI**^ and **v**_**3**_^**ST**^. This comparison shows that DTI performs poorly in measuring laminar orientation when compared to FI in fixed myocardium across the range of DTI imaging parameters investigated. Subsequently we compared DTI and ST determined sheetlet and myocyte orientation with each other directly.

### The Limitations of the DTI model

Physical studies have shown theoretical and experimental evidence that the DTI model has shortcomings which may limit its application in the measurement of cardiac orthotropy [22,23,41]. A monoexponential diffusion model is used to analyze the raw signals leading to DTI. This model envisages a single diffusion compartment in each image voxel. Importantly, it has been demonstrated in the ventricular myocardium of *ex vivo* perfused hearts that there may be more than one diffusion component per voxel (i.e. multiexponential diffusion, with more than one spin compartment) [22,23]. These diffusion components have been classified as a slow component (attributed to compartmentalization of intra- and extracellular (IC/EC) water pools) and a fast component (attributed to diffusion in the vascular space compartment combined with some IC diffusion) [22]. In imaging studies with short diffusion distances (low *b*-values, i.e. *b*-value < 1000s/mm^2^, where the b-value is defined below) the fast-component (vascular/IC) predominates, but this component still influences measurements at higher b-values. Blood vessels generally run parallel to local myocyte directions [42], and it has been suggested that the summation effect of slow and fast diffusion directions in the standard monoexponential DTI model is not an important practical consideration in the measurement of the local myocyte orientation. Indeed it has been shown that the local myocyte orientations calculated from the fast and slow components of diffusion are similar [22]. However, a consequence of two-component diffusion is that the proposed orthotropic diffusion may be complicated by non-orthotropic fast diffusion which could result in inaccurate measures of laminar orientation. Limitations of the diffusion model have not been addressed to date in direct validation studies of DTI myolaminar orientation measurement [21,40].

### b-value, diffusion gradient separation and DTI Imaging protocol used in this study

The b-value is a factor of diffusion weighted sequences which summarizes the influence of the sensitivity of the image to the diffusion gradients. As such the b-factor characterizes the extrinsic (sequence-based) imaging contrast determining factors and higher b-value corresponds to greater strength and duration of the diffusion gradients. With higher the b-value the stronger the influence of diffusion weighting on the image, but also the longer the gradients are applied, and the longer the duration of diffusion over which the tissue is probed.

1 $$ b = {\gamma}^2{\delta}^2\left(\varDelta \hbox{--} \delta /3\right)\left|\mathbf{g}\right|{}^2 $$

where γ is the proton gyromagnetic ratio (42 MHz/Tesla), **g** is the strength of the diffusion sensitizing gradient pulses, δ is the duration of the diffusion gradient pulses, Δ is the time of separation between diffusion gradient RF pulses. *b* has the units of s/mm^2^ . The b-value (*b*) was introduced and defined by (Le Bihan et al., 1986) [43]. The most appropriate b-value will therefore depend on the tissue structure (including its degree of anisotropy/orthotropy) [44] and on the tissue feature studied. In this study the sensitivity to b-value was explored in the range 500 to 2500 s/mm^2^, by variation of the strength of the diffusion gradient pulses (**g**) at fixed gradient pulse duration (δ = 3.6 ms) and fixed pulse separation (Δ = 11.5 ms), hence increasing the sensitivity to diffusion, but not changing the spatial scale over which the tissue anisotropy is probed by diffusion [45]. The gradient pulse separation has been termed mixing time and the optimal timing is also determined by the tissue structural feature of interest; generally shorter Δ is better tuned to measurement of finer spatial scale structural features and longer Δ is better tuned to measuring courser scale structural features. The optimal timings for gradient pulse separation for myocardial imaging have not been precisely determined but appropriate values depend critically on the tissue preparation and treatment prior to imaging. Fixation in formaldehyde shortens T_2_ and therefore TE and Δ must be shortened significantly to acquire sufficient signal [46,47]. Based on the T_2_ mapping of formaldehyde fixed rabbit myocardium a diffusion gradient separation of Δ = 12.6 ms was determined to be appropriate for balance between SNR and distinct DTI eigenvalues [47]. We based our diffusion gradient separation time for fixed rat myocardium on this value determined for rabbit as the same formaldehyde fixation approach was used and these species have globally similar cardiac myocyte and laminar structure [8]. The Δ = 11.5 ms used in this study is in the order of the Δ = 20.0 ms previously used to measure global differences in the systolic and diastolic orientation of sheetlets between rat hearts fixed in systole and fixed in diastole, a study in which there were statistically distinct λ_2_ and λ_3_ populations [35]. No published study has analyzed dependence of the accuracy of laminar measurement in fixed hearts on Δ. At 20°C, the diffusivity of free water is ~2.3×10^−3^ mm^2^/s [48], so in free water the root mean square displacement of a water molecule is ~7 μm when Δ = 11.5 ms. This displacement may be sub-optimal with respect to the spatial scale of sheetlets (~80 -120 μm thick according to [11]). Studies which have used DTI to measure laminar structure in un-fixed hearts have used diffusion gradient separation which is one to two orders of magnitude higher than values used in fixed heart studies, such as Δ = 400 ms in [21] for unfixed unperfused bovine myocardium DTI and Δ ≈ 1000 ms for *in vivo* human clinical myocardial DTI [45,49]. Kim et al. [50]. imaged *ex vivo* unperfused unfixed calf myocardium and showed that the difference between λ_1_ and λ_2_ and the difference between λ_1_ and λ_3_ increase when Δ is increased from 34 ms to 800 ms. This results in greater overall myocardial anisotropy indices at higher Δ, as measured by the fractional anisotropy index, the kurtosis index and the lattice index. The change in the eigenvalue ratios was observed between Δ = 34 ms and Δ = 100 ms, with minimal change above Δ = 100 ms. The Kim et. al. study, however, did not report any change in the ratio (global or local) of λ_2_ to λ_3_ and did not report any change in the orientation of **e**_**2**_^**DTI**^ or **e**_**3**_^**DTI**^ (the reported anisotropy measures are rotationally invariant and can’t assess change in orientation of eigenvectors). Although it has not been demonstrated in an experimental study it remains likely from theoretical considerations that the sensitivity of DTI to measuring sheetlet orientations (and hence the orientations of **e**_**2**_^**DTI**^ or **e**_**3**_^**DTI**^) will change with changing Δ. Similarly, it is possible that the low Δ imposed by the process of fixing tissue may limit the DTI measurement of sheetlet orientation in fixed myocardium, and that a wider range of structural features may be resolvable in un-fixed myocardium by the wider possible range of Δ values. Detailed direct 3D evaluation of the performance of DTI in measuring the sheetlets requires a structural method that can directly resolve sheetlet structure (i.e. by a method other than diffusion). Current applicable methods all require tissue fixation, and therefore only a limited range of Δ can be explored. Given these considerations, this study evaluates the performance of DTI in measuring sheetlet orientation in fixed hearts, which is a commonly performed experimental method used for myocardial structural measurement. The results of this assessment of DTI cannot (or can only to limited extent) be extrapolated to DTI sheetlet orientation measurement in un-fixed or *in vivo* tissue where longer Δ times are commonly employed.

### Sheetlet structure resolved within a voxel by the three methods

DTI and FLASH/ST both represent myocardial structure as a tensor and therefore both methods have inherent limitations in measuring myolaminar architecture in voxels which have 2 or more laminar orientations. The overall myolaminar orientation is simplified to a single laminar normal orientation vector. If a voxel contains two markedly different myolaminar orientations (e.g. sheetlets at 90° to each other, such as sheetlet-normal β” ~ −45° and β” ~ +45°) this will be quantified as a sheetlet-normal vector in a non-physical intermediate orientation (0° for this example case). This limitation is not inherent in the FI method, as a distribution of orientations is obtained for each 200 μm isotropic voxel, however in practice it is necessary to simplify this measure to a single (or small number) of local myolaminar orientations. This limitation of DTI and ST could be addressed by increasing resolution. There are literature reports on DTI in cardiac tissue with up to 100 μm isotropic resolution [51], but as voxel size is reduced imaging time is increased and SNR falls. The FLASH/ST and FI resolution can be increased by increasing the resolution of the FLASH to ~25 μm near-isotropic [11] or 25 μm (as used in [52]). Although some voxels have multiple sheetlet orientations (as shown in Figure 3), this is the minority and it can been seen by comparison with higher-resolution FLASH [11] that increasing FLASH voxel size does not resolve markedly greater complexity of laminar structure. In [11] we imaged the rat myocardium at higher FLASH resolution (25 × 25 × 34 μm) resolution and using 40 × histological magnification and the resultant images qualitatively show that increasing FLASH spatial resolution from 50 μm to 25 × 25 × 34 μm yields marginally greater myolaminar branching, and that further increasing resolution to 40 × histological magnification reveals minimal further branching.

### The nature of myolaminar structure

There has been ongoing controversy around the presence and nature of cardiac laminar architectural features [8]. Most surprisingly the debate around the presence or absence of laminar structure continues despite strong evidence from numerous imaging modalities. The mounting evidence includes conventional microscopic histology [11,40]; extended volume confocal microscopy [3,6,53]; confocal microscopy after myocardial optical clearing [54]; scanning electron microscopy [53], CMR [11], microCT [55], phase-contrast synchrotron x-ray imaging [56], direct myocardial video imaging and photography [11,12] and the orthotropic mechanical material properties of isolated myocardium [2,5,10]. The imaging and analysis in this study provides further evidence of the nature of laminar structure in the fixed heart. Further to [11] we show that laminar structure is near-universally present throughout the myocardium and that it exists as a highly-branching meshed structure of varying compactness, and is absent in some regions of the compact sub-epicardium. Laminar myocardial architecture was described by LeGrice et al. (1995) [12] as a branching structure, and in some of the subsequent literature this has been misinterpreted as single unique sheets of structure that have isolated spans across the myocardial wall [57]. This structural form was not proposed in LeGrice et al. (1995) [12] and indeed if the heart was structured in this way it would be prone to the mechanical separation of these laminae, and could be easily interactively dissected apart along these laminae, neither of which occurs. The 3D images in Figures 3, 4, 5 and 6 and Additional file 2: Movie 1 clearly show that myolaminar structure branches. As such there is no entity which is a single individual myolamina: the structure is orthotropic with preferential directions of sheetlet interstices and matching preferential directions of collagen orientation [6], but individual myocytes branch across the myolaminae in three-dimensions.

### Cardiac *ex vivo* contractile state

It is inevitable that fixation of hearts before imaging will result in some conformational change, such that the fixed structural state of the heart will not truly match an *in vivo* contractile state [8]. The fixed hearts are neither in a true physiological systolic or diastolic state.

### The application of the FI method

Using the FI method we showed that the quality of the FLASH is adequate to clearly define the sheetlet normal orientation through visualization, and we demonstrated that the ST method was appropriately applied. The raw FLASH images (as shown in Figure 6A and Additional file 2: Movie 1) provide a means of direct visualization and measurement of laminar architecture which can be isosurfaced to measure laminar orientations (Figure 3). A drawback of the FI method is that it is low-throughput and requires interactive segmentation, interactive visualization and interactive optimization of thresholds whereas DTI and ST/FLASH are both high-throughput automated methods. However the strength of the FI method is that each laminar normal orientation measurement had an accompanying 3D visualization of structure which was used to confirm that the method was functioning correctly and that orientation measurements reflected the underlying structure (as shown in Figure 3). The method can be used to measure laminar orientation, but cannot be used to measure local myocyte orientation, as the myocyte long axis is not resolved by the 50 × 50 × 50 μm^3^ spatial resolution of the FLASH. The reliability of DTI sheetlet orientation measurement (for the range of DTI imaging parameters explored in fixed myocardium, with fixed diffusion gradient separation time of Δ = 11.5 ms) compared to directly measured sheetlets varied depending on cardiac location: in the lateral ventricular wall the deviation angle (|∠**e**_**3**_^**DTI**^**n**^**FI**^|) was 23 ± 14° (median ± IQR) and in the septal wall deviation angle was 61 ± 53.4°. ST/FLASH is more reliable as in the lateral wall deviation angle was 9 ± 7° and in the septal wall 7 ± 9°. Furthermore, we show erroneous assignment of **e**_**3**_^**DTI**^ and **e**_**2**_^**DTI**^, in other words where the orientation assigned by DTI to the laminae sheetlet in-plane direction is actually much closer to the true laminar normal orientation. This was explored through assessment of the relative laminar eigenvalue magnitudes (ratios of laminar eigenvalues). DTI showed considerably less accuracy than ST when compared to the FI sheetlet normal orientation in both the lateral and septal ROI. In the septal ROI there was strong evidence of eigenvector missorting as the median difference between the DTI and FI laminar normals was 54.4 ± 47.3° and the deviation angle distribution was bimodal with the larger mode centered on 65° and including 65.1% of the lateral ROI voxels (Figure 7B). An absolute deviation angle of greater than 45° suggests eigenvector misassignment as angles >45° indicate that **e**_**2**_^**DTI**^ is closer to **n**^**FI**^ than **e**_**3**_^**DTI**^ (assuming that **e**_**1**_^**DTI**^ is close to the correct local myocyte orientation). The consequences of eigenvector misassignment are substantially deleterious to the accuracy of any imaging method that measures a tensor to encode structure, as the orthogonal system is rotated so as to produce a measure of the laminar-normal as different as possible to the correct values.

### Eigenvalue comparison

We showed that under the imaging parameter ranges explored in fixed myocardium there are greater grounds for sorting between the ST laminar eigenvectors than the DTI laminar eigenvectors. This was anticipated as: (i) ST is, from first principles, a method highly appropriate to quantifying laminar architecture in images where that structure is clearly visible on simple inspection; (ii) underlying myocardial diffusion has previously been shown to be complex, and the DTI model has limitations for describing this diffusion. We do not show that eigenvalue differences are insufficient to allow laminar measurement, but we show that there is little difference between the magnitudes of sheetlet eigenvalues and sheetlet-normal eigenvalues, and that **e**_**3**_^**DTI**^ is not close to **n**^**FI**^. The small difference between sheetlet eigenvalues and sheetlet-normal eigenvalues is a further indicator that DTI is a suboptimal measure of laminar orientation in fixed myocardium. This is further discussed in the Digital Supplement (section Eigenvalue Comparison).

### Direct comparison of DTI and ST measured myolaminar structure

The ST sheetlet angle and sheetlet normal angle maps largely recapitulate features described in the literature ([20,32,38,40,58,59] and discussed elsewhere [8]), and orientations can be seen to correspond to the FLASH image in Figure 6 and Additional file 4: Movie 2. We showed qualitatively and quantitatively that the degree of agreement between **e**_**3**_^**DTI**^ and **v**_**3**_^**ST**^ differed from region to region, and overall there was poor agreement. Likewise we showed qualitatively and quantitatively that the degree of agreement between **e**_**2**_^**DTI**^ and **v**_**2**_^**ST**^ differed from region to region, and overall there was poor agreement (worse than for the equivalent normal orientations). In this way we showed that the fixed-myocardium DTI secondary and tertiary eigenvector orientations are influenced by the underlying laminar architecture, but the assignment of these vectors to either orientation is not robust, and varies depending on cardiac location.

### Comparison between ST and FI

There is good agreement between the ST quantified laminar normal orientation and the FI quantified normal orientation (mean ± SD deviation 12.8° ± 13.2 for the combined equatorial ROI for STW = 3, DTW = 3). The mechanisms responsible for the systematic deviation between ST and the FI method based on FLASH have not been determined but it is likely associated with differences in the methodology used in the FI method to ST. ST is a tensor method so is influenced by the optimal orthotropic description of local contrast change (optimal fit of an orthogonal set of vectors with **v**_**3**_^**ST**^ in the direction normal to contrast change), **v**_**2**_^**ST**^ in the plane of local consistent contrast, and **v**_**1**_^**ST**^ in the direction of minimum contrast change. The FI method is only influenced by the local normal. It was noted that there was some eigenvalue misassignment in ST compared to FI. The probable reason for this is that unlike FI and DTI, in which just a single 200 × 200 × 200 μm^3^ voxel influences the local measured myolaminar normal orientation, in ST neighboring voxels also influence the tensor calculation (as discussed in detail in the Sensitivity Analysis section below). For at DTW = 3 each structure tensor component in the 200 × 200 × 200 μm^3^ representation has worked with intensities gathered from an effective 9 × 9 × 9 template at the original 50 μm resolution, i.e. around 450 × 450 × 450 μm^3^. However, the principal data weighting is at the center of that template. This will have minimal consequences when myolaminar structure is spatially conserved, but in regions of high-spatial change in laminar orientation then the neighbor influence will result in a different measure of laminar structure from ST/FLASH and from FI/FLASH.

### Comparison between DTI and ST local myocyte orientation

We showed that the ST myocyte α’ angle displays the familiar transmural rotation, observed circumferentially in the short-axis slice, and in the long-axis cuts. This myocyte helix angle has long been known from gross dissection, histology, and DTI [13] [8,60]. The myocyte transverse angle (α”) is generally described as closely following the left ventricle-short axis tangent orientation (i.e. this angle is ~0°). In Figure 6C α” deviates from this description as it has negative values, approaching −45° in the lateral sub-endocardium. These DTI images are not novel as many previous studies have measured and visualized the **e**_**1**_^**DTI**^ in this manner. However, the accompanying **v**_**1**_^**ST**^ images are novel and are striking in the degree of similarity between α’ and α” of **v**_**1**_^**ST**^ and of **e**_**1**_^**DTI**^. This has not previously been demonstrated. Despite disagreement between **e**_**3**_^**DTI**^ and **v**_**3**_^**ST**^ and **e**_**2**_^**DTI**^ and **v**_**2**_^**ST**^, there is remarkably good agreement between **e**_**1**_^**DTI**^ and **v**_**1**_^**ST**^. Our finding that DTI measures **m** well, but **s** and **n** poorly in fixed myocardium is consistent with the findings and conclusions of others [22,23], who found DTI reliable for measuring local myocyte orientation, but provided evidence that it was less reliable for measuring myolaminar orientation. The finding that ST measures **m** can seem counterintuitive as myocytes are not resolved by FLASH at 50 × 50 × 50 μm^3^ resolution as they have the approximate dimensions of 10 × 10 × 100 μm^3^ [8]. Indeed it is not possible to image whole rat hearts with conventional CMR imaging systems at sufficient resolution to be able to resolve individual myocytes. A resolution in the order of 3.5 × 3.5 × 3.5 μm^3^ would be required with good contrast and SNR, which is possible in small tissue blocks using phase-contrast synchrotron x-ray imaging [56] and is also obtainable in small volume MRI microscopy imaging [61] but not at the scale of the rat heart. Also, the small blood vessels cannot be discerned in the 50 μm isotropic resolution FLASH voxels (Figures 3, 4, 5, 6 and Additional file 2: Movie 1 and [11]). ST of the 50 × 50 × 50 μm^3^ FLASH data therefore depends on the tissue laminarity and orthotropy to measure the local myocyte orientation, and not myocyte orientation or blood vessel orientation. Given that ST is sensitive for measuring the image contrast changes associated with laminar orientation [11], then, if cardiac structure is genuinely close to orthotropic, it follows that **v**_**1**_^**ST**^ will be an accurate measure of local myocyte orientation. We show that **v**_**1**_^**ST**^ is a good approximation to the classically reported local myocyte orientation [13], and this provides further support for the orthotropic model of ventricular structure. We show that global myocyte orientation measured by DTI is well matched with the global myocyte orientation measured by ST. This is evidence which supports the use of DTI in measuring the local myocyte orientation.

It was observed that for both DTI and ST α” did not have a simple circumferential pattern, but rather had high negative values in the anterio-lateral myocardium (blue in Figure 6C and Figure 13). This pattern closely follows that reported for normal rat heart DTI in the literature [39]. It is likely largely a result of the cylindrical coordinate system used, which is based on a centroid optimized on the whole heart, rather than an individual slice and is a known source of error in α” [62], but it may to some degree be due to true variation of α”.

### Limitations

Here the term high-resolution is applied to FLASH CMR in the context of the whole heart geometry and with respect to clinical CMR, not with respect to the myocyte/sheetlet dimensions. The current study investigates conventional DTI on 200 μm isotropic voxels and b-values of 1000 s/mm^2^, with 6 or 12 gradient directions. Other Diffusion imaging models and approaches (such as High Angular Resolution Diffusion Imaging (HARDI) [63]) may perform better than DTI, and have the potential to provide greater sub-voxel information on sheetlet orientation. As DTI and FLASH both take several hours to perform imaging experiments are separated in time. However, the similarity between DTI and ST/FLASH determined local myocyte-orientations indicate that changes in structure between imaging experiments has been minimal. Both DTI and ST/FLASH produce a tensor which describes orthogonal cardiac structure. It is therefore implicit that in using these measures we take a starting assumption that myocardial structure is approximately orthotropic, and this has been demonstrated to be the case in the bulk of the ventricular myocardium [12]. The cylindrical coordinate system has greatest accuracy near the left ventricle equator, and has reduced accuracy approaching the left ventricle apex and base. The FI method as implemented in this study depends upon a manually optimized local tissue threshold.

This study explores DTI and FLASH in fixed myocardium perfused with gadolinium (Gadopentetate Dimeglumine). Many DTI studies have explored structure in fixed *ex vivo* cardiac tissue [20,32,38,40,58,60,64,65] and the findings in this study are relevant to the interpretation of past and future imaging of fixed myocardium. It is known that fixation results in structural changes including shrinkage and the opening up of sheetlet-interstices [8]. It is not known if sheetlet-interstices open equally on fixation and therefore the performance of FLASH, ST/CMR and DTI may differ when applied to *in vivo* or when applied to *ex vivo* physiologically perfused tissue, and the absolute values of sheetlet orientations may not reflect *in vivo* values. The high spatial resolution FLASH imaging requires ~18 hrs to image a rat heart and therefore requires cardiac fixation. As discussed in the section ‘*b-value, diffusion gradient separation and DTI Imaging protocol used in this study’* above, fixation shortens myocardial T_2_ and therefore imposes short DTI TE and diffusion gradient pulse separation (Δ) compared to parameter ranges possible in unfixed myocardium. A consequence is that more accurate measurement of sheetlet orientation may be possible in unfixed myocardium than can be achieved in fixed myocardium. Therefore our conclusions on the accuracy of DTI sheetlet orientation measurement cannot be extrapolated to DTI in unfixed or *in vivo* myocardium.

## Conclusions

DTI and ST both produce tensors whose eigenvectors correspond to a greater-or-lesser degree with the cardiac orthotropic structural axes. DTI and ST predict globally similar myocyte orientations, and this evidence supports using DTI to measure local myocyte orientation. DTI produces smoother local myocyte orientation maps and is faster for imaging local myocyte orientation but with appropriate regularization, ST is likely to be useful for this purpose also. In fixed myocardium ST is a better measure of myolaminar orientation than DTI over the parameter ranges explored. The reliability of DTI sheetlet orientation measurement compared to directly measured sheetlets was low or markedly low depending on cardiac location and we showed that poor DTI performance over this parameter range could be a consequence of poor laminar eigenvector assignment. Sensitivity analysis showed that overall DTI sensitivity to time post fixation is low, to b-value is moderate (with fixed diffusion gradient separation time of Δ = 11.5 ms), and to number of diffusion directions is low. Optimal DTI imaging parameters were b = 1000 mm/s^2^ and 12 diffusion-directions with time post fixation up to 72 hours not being an important factor. ST was not sensitive to image processing parameters. FLASH and ST requires more time (~1 day) compared to DTI (~3 hours). However, FLASH directly resolves myolaminae and FLASH/ST more accurately quantifies the orientation of these structures. We conclude that the FLASH/ST framework is reliable, robust and the preferred option for myolaminar measurement in fixed myocardium. DTI has an important role for local myocyte orientation measurement, and a method could be developed for combining the optimal characteristics of ST and of DTI. The methods developed and assessed in this study will be useful in future development and refinement of diffusion CMR of cardiac structure, both *in vivo* and *ex vivo*. Future studies are required to quantify the accuracy of structural orientation measurement by DTI in unfixed myocardium (either *in vivo* or with *ex vivo* perfused hearts) against direct 3D imaging measurements.

## Additional files

Additional file 1:
**Digital Supplement.** Supplemental Methods, Results and Discussion. Additional file 2:
**Movie 1.** Movie 1 - visualization of the 3D laminar architecture of a rat heart. The segmented FLASH image is shown after cropping off the cardiac base with the left ventricle and right ventricle labelled on the short-axis surface, and a cyan contour outlining the epicardial and endocardial short-axis boundaries. Sheetlets (black) and the sheetlet-interstices (white) are visible on the short-axis surface. The cardiac volume is first rotated through 360°. The right-anterior half-heart is faded out leaving the left-posterior half-heart, and the cropping plane is outlined with a cyan square. The full cardiac volume is then rotated around a static cropping plane (the red box), showing on the long-axis cropped surface a movie rotating through the entire internal sheetlet structure of the left ventricle and right ventricle. This cropping process can be confusing on first viewing of the movie, so we recommend viewing the movie several times, and pausing and advancing it frame by frame to understand the process and to clearly visualize the myocardial laminar structure. The left ventricle labels and the position of the original cropping plane (cyan, rotating) help to orientate the myocardial tissue present in the frame. The myocardium present and the cut from the image is described above the cardiac volume. The tissue rotating through the static cropping plane is first shown with a transparent epicardial surface coloring for 360° and rotation is repeated for 360° with no epicardial shading. Small artefacts are visible in the CMR image but these detract little from the visualization of whole heart laminar structure (there is bright artefact where the epicardial surface is touching the imaging container on the anterior and posterior epicardium and dark susceptibility artefact in small areas of myocardium contrast loss in the image particularly in the right ventricle). T1W is T1-weighted FLASH; FLASH: fast low angle shot; LV: left ventricle; RV: right ventricle; SA: short-axis; LA: long-axis; lat.: lateral; ant.: anterior; post.: posterior. Additional file 3: Figure DS1. Visualization of the difference between ST and DTI putative sheetlet-in-plane orientation vectors and angles. **A** - angle between the DTI (6-direction) and ST putative sheetlet in-plane vectors, which are colored according to the 0° to +90° scale shown. **B** - the ST and DTI putative sheetlet in-plane elevation (β’) and sheetlet in-plane transverse(β’’) angle maps, which are colored according to the -90° to +90° scale shown. DTI: Scan #1, 6-direction, b = 1000 s/mm^2^; ST: Scan #8, DTW = 3, STW = 3. FLASH: fast low angle shot; ST: structure tensor of FLASH data; DTI: diffusion tensor magnetic resonance imaging; DTW: derivative template width STW: smoothing template width. The symbols for vectors and derived angles are defined in Table 2. Additional file 4: Movie 2. Movie 2 - visualization of the putative sheetlet-normal orientations from ST and DTI through the volume of the rat heart. The treatment and presentation of the cardiac volume is as described for Movie 1 and familiarization with the views presented in Movie 1 is essential before viewing Movie 2. The cardiac volume is false-colored (according to the color scale shown) for the angles β’ (helix) and β” (transverse) of the putative sheetlet-normal orientation. The epicardial surface is shaded dark gray and the left ventricle endocardial surface is shaded light gray. The movie has two successive labelled 360° rotations of the cardiac geometry through a static cropping plane (the red square) showing the following putative sheetlet-normal angles: In the first rotation, on the left the putative sheetlet elevation angle (β’) from ST (i.e. β’v_3_^ST^) and on the right the putative sheetlet elevation angle (β’) from DTI (i.e. β’e_3_^DTI^); In the second rotation, on the left the putative sheetlet transverse angle (β”) from ST (i.e. β”v_3_^ST^) and on the right the putative sheetlet transverse angle (β”) from DTI (i.e. β”e_3_^DTI^). The tissue presented and the cut from the image are described above the cardiac volume. DTI: parameters: 12-direction, b = 1000 s/mm^2^; ST parameters: DTW = 3, STW = 3. FLASH: fast low angle shot; ST: structure tensor of FLASH data; DTI: diffusion tensor magnetic resonance imaging; DTW: derivative template width STW: smoothing template width. The symbols for vectors and derived angles are defined in Table 2. Additional file 5: Figure DS5. Summary sensitivity analysis data, referenced to the ground truth, for all scans for all ROI. The mean and standard deviation of |∠**[v**_**3**_^**ST**^ **n** ^**FI**^| or |∠**[e**_**3**_^**DTI**^ **n** ^**FI**^| are shown for the lateral, septal, anterior and posterior ROI. The number following the # is the scan number as defined in Table 1. ^a^ – scan # 8, T1W FLASH scan with processing parameters STW = 3, DTW = 3. ^b^ – scan # 8, FLASH T1W FLASH scan with processing parameters STW = 5, DTW = 5. FLASH: fast low angle shot; ST: structure tensor of FLASH data; DTI: diffusion tensor magnetic resonance imaging; DTW: derivative template width STW: smoothing template width. The symbols for vectors and derived angles are defined in Table 2. Additional file 6: Figure DS2. The ST and DTI putative sheetlet-normal angles are compared for 5 rat hearts. The **v**_**3**_^**ST**^ and **e**_**3**_^**DTI**^ transverse (β’’) angle maps of an equatorial short-axis slice are colored according to the -90° to +90° scale. Regions of similar and differing laminar normal orientation are shown in the magenta and black boxes respectively. The transmural orange line on the FLASH images indicates the transmural span quantified in Figure 14. DTI: Scan #1, 6-direction, b = 1000 s/mm^2^; ST: Scan #8, DTW = 3, STW = 3. FLASH: fast low angle shot; ST: structure tensor of FLASH data; DTI: diffusion tensor magnetic resonance imaging; DTW: derivative template width STW: smoothing template width. The symbols for vectors and derived angles are defined in Table 2. The associated angle maps for the **v**_**3**_^**ST**^ and **e**_**3**_^**DTI**^ elevation (β’) angle are in Figure 9. Additional file 7: Figure DS3. Equatorial lateral ROI distributions of angle differences between the ST and DTI vectors/orientation angles. **A** – deviation angles |∠**v**_**3**_^**ST**^ **e**_**3**_^**DTI**^| and |∠**v**_**2**_^**ST**^ **e**_**2**_^**DTI**^| are shown, alongside the corresponding distributions of |∠β’**v**_**3**_^**ST**^β’**e**_**3**_^**DTI**^|, |∠β”**v**_**3**_^**ST**^β”**e**_**3**_^**DTI**^|, |∠β’**v**_**2**_^**ST**^β’**e**_**2**_^**DTI**^|, |∠β”**v**_**2**_^**ST**^β”**e**_**2**_^**DTI**^|. **B** – deviation angles |∠**v**_**1**_^**ST**^ **e**_**1**_^**DTI**^| and |∠**v**_**1**_^**ST**^ **e**_**1**_^**DTI**^| are shown, alongside the corresponding distributions of |∠α’**v**_**1**_^**ST**^α’**e**_**1**_^**DTI**^|, |∠α”**v**_**1**_^**ST**^α”**e**_**1**_^**DTI**^|, |∠α’**v**_**1**_^**ST**^α’**e**_**1**_^**DTI**^|, |∠α”**v**_**1**_^**ST**^α”**e**_**1**_^**DTI**^|. DTI: Scan #1, 6-direction, b = 1000 s/mm^2^; ST: Scan #8; DTW = 3; STW = 3. FLASH: fast low angle shot; ST: structure tensor of FLASH data; DTI: diffusion tensor magnetic resonance imaging; DTW: derivative template width STW: smoothing template width; MAD: median absolute deviation; ROI: region(s) of interest. The symbols for vectors and derived angles are defined in Table 2. The corresponding distributions for the equatorial septal ROI are in Figure 10. Additional file 8: Figure DS4. Differences between ST and DTI vary depending on cardiac location and are stable over time. Results are presented by region (lateral, septal) showing the deviation between the ST and the corresponding DTI eigenvector orientations pairs (of **v**_**1**_^**ST**^ **e**_**1**_^**DTI**^ , **v**_**2**_^**ST**^ **e**_**2**_^**DTI**^ and **v**_**3**_^**ST**^ **e**_**3**_^**DTI**^), and the difference between the associated vector elevation and transverse angles. Side **A** (left) of each histogram are angles from comparison of ST to a DTI image taken in the 2 hours BEFORE the FLASH (Scan #7). Side **B** are from comparison of ST to a DTI image taken in the 2 hours AFTER the FLASH (Scan #9). DTI: 6-direction, b = 1000 s/mm^2^; ST: Scan #8, DTW = 3, STW = 3. FLASH: fast low angle shot; ST: structure tensor of FLASH data; DTI: diffusion tensor magnetic resonance imaging; DTW: derivative template width STW: smoothing template width. The symbols for vectors and derived angles are defined in Table 2. The corresponding distributions for the lateral and septal ROI are in Figure 11. Additional file 9: Movie 3. Movie 3 - visualization of the putative myocyte orientations from ST and DTI through the volume of the rat heart. The treatment and presentation of the cardiac volume is as described for Movie 1&2 and familiarization with the views presented in these movies is essential before viewing Movie 3. The cardiac volume is false-colored (according to the color scale shown) for the angles α’ and α” of the putative myocyte orientation. The epicardial surface is shaded dark gray and the left ventricle endocardial surface is shaded light gray. The movie has two successive labelled 360° rotations of the cardiac geometry through a static cropping plane (the red square) showing the following putative myocyte angles: In the first rotation, on the left the putative myocyte helix angle (α’) from ST (i.e. α’v_31_^ST^) and on the right the putative myocyte helix angle (α’) from DTI (i.e. α’e_1_^DTI^); In the second rotation, on the left the putative myocyte transverse angle (α”) for ST (i.e. α”v_1_^ST^) and on the right the putative myocyte transverse angle (α”) for DTI (i.e. α”e_1_^DTI^). The tissue presented and the cut from the image are described above the cardiac volume. DTI: parameters: 12-direction, b = 1000 s/mm^2^; ST parameters: DTW = 3, STW = 3. FLASH: fast low angle shot; ST: structure tensor of FLASH data; DTI: diffusion tensor magnetic resonance imaging; DTW: derivative template width STW: smoothing template width. The symbols for vectors and derived angles are defined in Table 2. Additional file 10: Figure DS6. Sensitivity of the deviation between **v**_**3**_^**ST**^ or **e**_**3**_^**DTI**^ and **n** ^**FI**^ to imaging/image processing parameters. This was explored in the from the four ROI (lateral, septal, anterior, posterior), showing the mean and standard deviation of |∠**[v**_**3**_^**ST**^ **n** ^**FI**^| or |∠**[e**_**3**_^**DTI**^ **n** ^**FI**^|. **A** – sensitivity of **e**_**3**_^**DTI**^ to time post-fixation from 2 to 71 hours. **B** – sensitivity of **e**_**3**_^**DTI**^ to b-value from 500 to 2500 s/mm^2^. **C** – sensitivity of **e**_**3**_^**DTI**^ to number of diffusion directions from 6 to 12 directions. **D** – sensitivity of **v**_**3**_^**ST**^ to the DTW and STW. ST: Scan #8. FLASH: fast low angle shot; ST: structure tensor of FLASH data; DTI: diffusion tensor magnetic resonance imaging; DTW: derivative template width STW: smoothing template width. The symbols for vectors and derived angles are defined in Table 2.

## References

[1] LeGrice IJ, Pope AJ, Sands GB, Whalley G, Doughty RN, Smaill BH (2012). Progression of myocardial remodeling and mechanical dysfunction in the spontaneously hypertensive rat. Am J Physiol Heart Circ Physiol.

[2] Hooks DA, Trew ML, Caldwell BJ, Sands GB, LeGrice IJ, Smaill BH (2007). Laminar arrangement of ventricular myocytes influences electrical behavior of the heart. Circ Res.

[3] Sands GB, Gerneke DA, Hooks DA, Green CR, Smaill BH, Legrice IJ (2005). Automated imaging of extended tissue volumes using confocal microscopy. Microsc Res Tech.

[4] Trew ML, Caldwell BJ, Sands GB, Hooks DA, Tai DC, Austin TM, Legrice IJ, Pullan AJ, Smaill BH (2006). Cardiac electrophysiology and tissue structure: bridging the scale gap with a joint measurement and modelling paradigm. Exp Physiol.

[5] Caldwell BJ, Trew ML, Sands GB, Hooks DA, LeGrice IJ, Smaill BH (2009). Three distinct directions of intramural activation reveal nonuniform side-to-side electrical coupling of ventricular myocytes. Circ Arrhythm Electrophysiol.

[6] Pope AJ, Sands GB, Smaill BH, LeGrice IJ (2008). Three-dimensional transmural organization of perimysial collagen in the heart. Am J Physiol Heart Circ Physiol.

[7] Hales PW, Schneider JE, Burton RA, Wright BJ, Bollensdorff C, Kohl P (2012). Histo-anatomical structure of the living isolated rat heart in two contraction states assessed by diffusion tensor MRI. Prog Biophys Mol Biol.

[8] Gilbert SH, Benson AP, Li P, Holden AV (2007). Regional localisation of left ventricular sheet structure: integration with current models of cardiac fibre, sheet and band structure. Eur J Cardiothorac Surg.

[9] Costa KD, Takayama Y, McCulloch AD, Covell JW (1999). Laminar fiber architecture and three-dimensional systolic mechanics in canine ventricular myocardium. Am J Physiol.

[10] Dokos S, Smaill BH, Young AA, LeGrice IJ (2002). Shear properties of passive ventricular myocardium. Am J Physiol Heart Circ Physiol.

[11] Gilbert SH, Benoist D, Benson AP, White E, Tanner SF, Holden AV, Dobrzynski H, Bernus O, Radjenovic A (2012). Visualization and quantification of whole rat heart laminar structure using high-spatial resolution contrast-enhanced MRI. Am J Physiol Heart Circ Physiol.

[12] LeGrice IJ, Smaill BH, Chai LZ, Edgar SG, Gavin JB, Hunter PJ (1995). Laminar structure of the heart: ventricular myocyte arrangement and connective tissue architecture in the dog. Am J Physiol.

[13] Streeter DD, M. BR (1979). Vol. I. The heart. Section 2: The cardiovascular system: Gross morphology and fiber geometry of the heart. *Handbook of Physiology Volume* 1.

[14] Colli Franzone P, Guerri L, Taccardi B (1993). Potential distributions generated by point stimulation in a myocardial volume: simulation studies in a model of anisotropic ventricular muscle. J Cardiovasc Electrophysiol.

[15] Trayanova NA (2011). Whole-heart modeling: applications to cardiac electrophysiology and electromechanics. Circ Res.

[16] Wang VY, Lam HI, Ennis DB, Cowan BR, Young AA, Nash MP (2009). Modelling passive diastolic mechanics with quantitative MRI of cardiac structure and function. Med Image Anal.

[17] Eriksson TS, Prassl AJ, Plank G, Holzapfel GA (2013). Modeling the dispersion in electromechanically coupled myocardium. International journal for numerical methods in biomedical engineering.

[18] Hsu EW, Muzikant AL, Matulevicius SA, Penland RC, Henriquez CS (1998). Magnetic resonance myocardial fiber-orientation mapping with direct histological correlation. Am J Physiol.

[19] Gilbert SH, Benson AP, Li P, Holden AV, Sachse FBSG (2007). Visualisation of dog myocardial structure from diffusion tensor magnetic resonance imaging: The paradox of uniformity and variability. *Functional Imaging and Modeling of the Heart, Proceedings. Volume* 4466.

[20] Scollan DF, Holmes A, Zhang J, Winslow RL (2000). Reconstruction of cardiac ventricular geometry and fiber orientation using magnetic resonance imaging. Ann Biomed Eng.

[21] Tseng WY, Wedeen VJ, Reese TG, Smith RN, Halpern EF (2003). Diffusion tensor MRI of myocardial fibers and sheets: correspondence with visible cut-face texture. J Magn Reson Imaging.

[22] Hsu EW, Buckley DL, Bui JD, Blackband SJ, Forder JR (2001). Two-component diffusion tensor MRI of isolated perfused hearts. Magn Reson Med.

[23] Forder JR, Bui JD, Buckley DL, Blackband SJ (2001). MR imaging measurement of compartmental water diffusion in perfused heart slices. Am J Physiol Heart Circ Physiol.

[24] Kohler S, Hiller KH, Waller C, Jakob PM, Bauer WR, Haase A (2003). Visualization of myocardial microstructure using high-resolution T*2 imaging at high magnetic field. Magn Reson Med.

[25] Jähne B (2005). Digital image processing.

[26] Farid H, Simoncelli EP (2004). Differentiation of Discrete Multidimensional Signals. IEEE Trans Image Process.

[27] Gilbert SH, Sands GB, LeGrice IJ, Smaill BH, Bernus O, Trew ML (2012). A framework for myoarchitecture analysis of high resolution cardiac MRI and comparison with diffusion Tensor MRI. Conference Proceedings.

[28] Gilbert SH, Trew ML, Smaill BH, Radjenovic A, Bernus O. Measurement of Myocardial Structure: 3D Structure Tensor Analysis of High Resolution MRI Quantitatively Compared to DT-MRI. Statistical Atlases and Computational Models of the Heart 2012. In in Lect Notes Comput Sc. 2012;7746:207–14.

[29] Papadakis NG, Xing D, Huang CL, Hall LD, Carpenter TA (1999). A comparative study of acquisition schemes for diffusion tensor imaging using MRI. J Magn Reson.

[30] Benson AP, Gilbert SH, Li P, Newton SM, Holden AV (2008). Reconstruction and Quantification of Diffusion Tensor Imaging-Derived Cardiac Fibre and Sheet Structure in Ventricular Regions used in Studies of Excitation Propagation. Mathematical Modelling Nat Phenomena.

[31] Marchand P, Marmet L (1983). Binomial smoothing filter: A way to avoid some pitfalls of least-squares polynomial smoothing. Rev Sci Instrum.

[32] Helm PA, Tseng HJ, Younes L, McVeigh ER, Winslow RL (2005). Ex vivo 3D diffusion tensor imaging and quantification of cardiac laminar structure. Magn Reson Med.

[33] Yoo TS, Ackerman MJ, Lorensen WE, Schroeder W, Chalana V, Aylward S, Metaxas D, Whitaker R (2002). Engineering and algorithm design for an image processing Api: a technical report on ITK–the Insight Toolkit. Stud Health Technol Inform.

[34] Hsu EW (2002). Myocardial Fiber Orientation Mapping via MR Diffusion Tensor Imaging. *Joint EMBS-BMES conference*; *Oct*.

[35] Chen J, Liu W, Zhang H, Lacy L, Yang X, Song SK, Wickline SA, Yu X (2005). Regional ventricular wall thickening reflects changes in cardiac fiber and sheet structure during contraction: quantification with diffusion tensor MRI. Am J Physiol Heart Circ Physiol.

[36] Fisher R (1953). Dispersion on a Sphere. Proc R Soc Lond A Math Phys Sci.

[37] Scheer P, Sverakova V, Doubek J, Janeckova K, Uhrikova I, Svoboda P (2012). Basic values of M-mode echocardiographic parameters of the left ventricle in outbreed Wistar rats. Vet Med.

[38] Helm PA, Younes L, Beg MF, Ennis DB, Leclercq C, Faris OP, McVeigh E, Kass D, Miller MI, Winslow RL (2006). Evidence of structural remodeling in the dyssynchronous failing heart. Circ Res.

[39] Chen J, Song SK, Liu W, McLean M, Allen JS, Tan J, Wickline SA, Yu X (2003). Remodeling of cardiac fiber structure after infarction in rats quantified with diffusion tensor MRI. Am J Physiol Heart Circ Physiol.

[40] Kung GL, Nguyen TC, Itoh A, Skare S, Ingels NB, Miller DC, Ennis DB (2011). The presence of two local myocardial sheet populations confirmed by diffusion tensor MRI and histological validation. J Magn Reson Imaging.

[41] Hsu EW, Xue R, Holmes A, Forder JR (1998). Delayed reduction of tissue water diffusion after myocardial ischemia. Am J Physiol.

[42] Bassingthwaighte JB, Yipintsoi T, Harvey RB (1974). Microvasculature of the dog left ventricular myocardium. Microvasc Res.

[43] Le Bihan D, Breton E, Lallemand D, Grenier P, Cabanis E, Laval-Jeantet M (1986). MR imaging of intravoxel incoherent motions: application to diffusion and perfusion in neurologic disorders. Radiology.

[44] Kingsley PB, Monahan WG (2004). Selection of the optimum b factor for diffusion-weighted magnetic resonance imaging assessment of ischemic stroke. Magn Reson Med.

[45] Scott AD, Ferreira PF, Nielles-Vallespin S, Gatehouse P, McGill LA, Kilner P, et al. Optimal diffusion weighting for in vivo cardiac diffusion tensor imaging. *Magn Reson Med* 2014, In Press. 10.1002/mrm.25418 25154715

[46] Thickman DI, Kundel HL, Wolf G (1983). Nuclear magnetic resonance characteristics of fresh and fixed tissue: the effect of elapsed time. Radiology.

[47] Holmes AA, Scollan DF, Winslow RL (2000). Direct histological validation of diffusion tensor MRI in formaldehyde-fixed myocardium. Magn Reson Med.

[48] Wu Y, Tepper HL, Voth GA (2006). Flexible simple point-charge water model with improved liquid-state properties. J Chem Phys.

[49] Ferreira PF, Kilner PJ, McGill LA, Nielles-Vallespin S, Scott AD, Ho SY, McCarthy KP, Haba MM, Ismail TF, Gatehouse PD (2014). In vivo cardiovascular magnetic resonance diffusion tensor imaging shows evidence of abnormal myocardial laminar orientations and mobility in hypertrophic cardiomyopathy. J Cardiovasc Magn Reson.

[50] Kim S, Chi-Fishman G, Barnett AS, Pierpaoli C (2005). Dependence on diffusion time of apparent diffusion tensor of ex vivo calf tongue and heart. Magn Reson Med.

[51] Jiang Y, Pandya K, Smithies O, Hsu EW (2004). Three-dimensional diffusion tensor microscopy of fixed mouse hearts. Magn Reson Med.

[52] Bishop MJ, Plank G (2012). The role of fine-scale anatomical structure in the dynamics of reentry in computational models of the rabbit ventricles. J Physiol.

[53] Young AA, Legrice IJ, Young MA, Smaill BH (1998). Extended confocal microscopy of myocardial laminae and collagen network. J Microsc.

[54] Smith RM, Matiukas A, Zemlin CW, Pertsov AM (2008). Nondestructive optical determination of fiber organization in intact myocardial wall. Microsc Res Tech.

[55] Haibo N, Castro SJ, Stephenson RS, Jarvis JC, Lowe T, Hart G, Boyett MR, Henggui Z (2013). Extracting myofibre orientation from micro-CT images: An optimisation study. Computing in Cardiology Conference (CinC).

[56] Varray F, Wang L, Fanton L, Zhu Y-M, Magnin I, Ourselin S, Rueckert D, Smith N (2013). High Resolution Extraction of Local Human Cardiac Fibre Orientations. *Functional Imaging and Modeling of the Heart. Volume* 7945.

[57] Lunkenheimer P, Niederer P, Sanchez-Quintana D, Murillo M, Smerup M (2013). Models of Ventricular Structure and Function Reviewed for Clinical Cardiologists. J Cardiovasc Trans Res.

[58] Helm P, Beg MF, Miller MI, Winslow RL (2005). Measuring and mapping cardiac fiber and laminar architecture using diffusion tensor MR imaging. Ann N Y Acad Sci.

[59] Beg MF, Helm PA, McVeigh E, Miller MI, Winslow RL (2004). Computational cardiac anatomy using MRI. Magn Reson Med.

[60] Hsu EW, Henriquez CS (2001). Myocardial Fiber Orientation Mapping Using Reduced Encoding Diffusion Tensor Imaging. J Cardiovasc Magn Reson.

[61] Lee SC, Kim K, Kim J, Lee S, Han Yi J, Kim SW, Ha KS, Cheong C (2001). One micrometer resolution NMR microscopy. J Magn Reson.

[62] Geerts L, Bovendeerd P, Nicolay K, Arts T (2002). Characterization of the normal cardiac myofiber field in goat measured with MR-diffusion tensor imaging. Am J Physiol Heart Circ Physiol.

[63] Dierckx H, Benson AP, Gilbert SH, Ries ME, Holden AV, Verschelde H, et al. Intravoxel Fibre Structure of the Left Ventricular Free Wall and Posterior Left-Right Ventricular Insertion Site in Canine Myocardium Using Q-Ball Imaging. Functional Imaging and Modeling of the Heart, Proceedings. In Lect Notes Comput Sc. 2009;5528:495–504.

[64] Scollan DF, Holmes A, Winslow R, Forder J (1998). Histological validation of myocardial microstructure obtained from diffusion tensor magnetic resonance imaging. Am J Physiol.

[65] Holmes AA, Scollan DF, Winslow RL (2000). Direct histological validation of diffusion tensor MRI in formaldehyde-fixed myocardium. Magn Reson Med.

