# Supplementary material for: Comparison of diffusion tensor imaging by cardiovascular magnetic resonance and gadolinium enhanced 3D image intensity approaches to investigation of structural anisotropy in explanted rat hearts
Source: J Cardiovasc Magn Reson. 2015 Apr 29;17(1):31. doi: 10.1186/s12968-015-0129-x (PMC4414435; doi:10.1186/s12968-015-0129-x)
Supplement: Additional file 1: — Digital Supplement. Supplemental Methods, Results and Discussion. [file 12968_2015_129_MOESM1_ESM.docx]

^[[1]](#footnote-1)^

***Digital Supplement***

**METHODS**

**Heart Preparation and Perfusion Fixation**

Hearts were perfused in Langendorff mode at 7 ml/min with HEPES-Tyrode solution for 5 min to allow beating to resume and to assist clearing of blood. The HEPES-Tyrode solution was composed of (in mM): 130 NaCl; 5.4 KCl; 0.4 NaH_2_PO_4_; 1.4 MgCl_2_,6H_2_O; 5 HEPES; 10 glucose; 20 taurine; 10 creatine, pH adjusted to 7.4 with NaOH, 0.75mM CaCl_2_ and containing 0.1% Heparin. Cardiac contraction was then prevented by perfusion with Tyrode solution containing 0 mM CaCl_2_ and 10mM 2,3-butanedione monoxime (BDM) for 5 min Next, the hearts were simultaneously perfused with MRI contrast agent and fixative for 20 min, using Tyrode solution containing 0 mM CaCl_2_, 4% formaldehyde, and 0.1% Gd-DTPA (Dimeglumine gadopentetate, Magnevist, Bayer Schering Pharma). The hearts were then removed from the perfusion apparatus and stored 2 hours at 20°C in the contrast/fixative solution before imaging. Tissue contrast enhancement is a result of differential Gd-DTPA uptake at steady state rather than non-equilibrium dynamic change in Gadolinium levels as in early and late gadolinium enhancement studies [[1](#_ENREF_1)].

**Circular Statistics**

Circular statistics were used for all periodic angular data as described in [[2](#_ENREF_2)]. **K** - (Kappa) was used to characterize the circular dispersion of myolaminar orientation data. **K** is a measure of dispersion of circular and spherical data (sometimes reported as **K** ^-1^) such that as dispersion tends towards 0, **K** → ∞ (and **K** ^-1^ → 0).

**RESULTS**

**Comparison of ST and DTI myocardial structural measurements**

Data for additional ROI and additional derived sheetlet angles are provided in this supplement. The main manuscript Fig. 6 is supplemented by Fig. DS1 (Additional file 3) which shows the angular difference between ST and DTI putative sheetlet in-plane vectors and shows the β’ and β’’ angles for the putative sheetlet in-plane vectors. Main manuscript Fig. 9 is supplemented by Fig. DS2 (Additional file 6) which shows comparative equatorial putative sheetlet-normal β’’ maps for 5 rat hearts. Main manuscript Fig. 10 is supplemented by Fig. DS3 (Additional file 7) which shows equatorial lateral ROI distributions of angle differences between the ST and DTI vectors/orientation angles. Main manuscript Fig. 11 is supplemented by Fig. DS4 (Additional file 8) which shows that differences between ST and DTI vary depending on cardiac location and are stable over time, with data from the posterior and anterior ROI.

**DTI and ST Sensitivity Analysis**

A series of imaging experiments were carried out to explore sensitivity of ST and DTI to imaging parameters and the results of this are summarized in Supplementary Table DS1. Fig. DS5 (Additional file 5) shows summary sensitivity analysis data, referenced to the ground truth, for all scans for all ROI. Sensitivity analysis showed that in the *ex vivo* heart the sensitivity to time post-fixation between 3:50 and 72:45 (h:min) of **e_1_^DTI^**, **e_2_^DTI^** and **e_3_^DTI^** is low (<5°, Fig. DS6A, Additional file 10). There is only marginal difference between **e_1_^DTI^**, **e_2_^DTI^**, and **e_3_^DTI^** values obtained closest to the time of heart fixation and those obtained longer after the time of heart fixation. Sensitivity to b-value between 500 to 2500 s/mm^2^ (with b-value varied by changing gradient pulse amplitude at fixed pulse separation Δ = 11.5 ms) of **e_3_^DTI^** is low when referenced to the **n^FI^** (Fig. DS6B, Additional file 10). Sensitivity to b-value between 500 to 2500 s/mm^2^ of (i) of **e_1_^DTI^** is low (<5°); (ii) of **e_2_^DTI^** is moderate (5-10°); and, (iii) of **e_3_^DTI^** is moderate (5-10°) when referenced to the corresponding ST eigenvectors. The optimal b-value is 1000 s/mm^2^ (Fig. DS6B, Additional file 10). Sensitivity to number of diffusion directions between 6 and 12: (i) of **e_1_^DTI^** is close to 0°; (ii) of **e_2_^DTI^** is low (<5°); and, (iii) of **e_3_^DTI^** is low (<5°) (Fig. DS6C, Additional file 10). The marginally optimal number of diffusion directions is 12. Sensitivity of **e_1_^ST^** to ST image processing parameters STW and DTW is close to 0° (Fig. DS6D, Additional file 10). When ROI are explored individually higher sensitivity to individual parameters are revealed in some ROI. Although DTI myostructural measurement has low sensitivity to parameters, as observed previously, irrespective of the time (between 3:50 and 72:45 (h:min)), irrespective of b-value (from 500 to 2500 s/mm^2^), and irrespective of number of diffusion directions (between 6 and 12): (i) the deviation angle of **e_3_^DTI^** remains large with respect to **n^FI^** and with respect to **v_3_^ST^**; (ii) the deviation angle of **e_2_^DTI^** remains large with respect to **v_2_^ST^**; and, (iii) the deviation angle of **e_1_^DTI^** remains small with respect to **v_1_^ST^**.

Stability of the DTI structural orientation measurements with respect to the time post-fixation are further demonstrated in primary manuscript Fig. 11 and Fig. DS4 (Additional file 8). In these figures two histograms are presented for each of the septal, lateral, anterior and posterior ROIs. The left histogram shows the mean and SD of sheetlet and myocyte orientation deviation angles calculated from the reference 6-direction DTI scan (#7 in primary manuscript Table 1, ending at 21h08m) before the FLASH scan (#8, ending at 39h20m). The right histogram shows the same angles, but calculated from DTI scan #9 (ending at 41h10m), after the same FLASH scan (#8). The data in the left and right histograms are therefore separated by the 18h12m duration of the FLASH scan. The paired histograms are near-identical and minimal or no time-associated changes observed between the 9 corresponding angle deviations. Considering all 9 deviation angles the maximum change over 18 hours is -1.6° (for |∠β’**e_2_^DTI^**β’**v_2_^ST^**|), the maximum change in SD is -1.3° (for |∠**e_3_^DTI^v_3_^ST^**|, the average change in mean difference is -1.0° and the average change in the deviation angle SD is +0.8°. In summary, sensitivity of whole ROI **e_1_^DTI^**, **e_2_^DTI^** and **e_3_^DTI^** to time is low, as the maximum deviation measured over the time scale in this series of analyses is 7.0°. This analysis shows that any differences between the myocyte, sheet, and sheet normal orientation distributions based on structural change over time (as reported by[[3](#_ENREF_3)]) are small.

**DISCUSSION**

**Discussion of the Results of Other Validation Studies**

The first histological study which reported to validate DTI sheetlet measurement was carried out by Tseng et al. (2003) [[4](#_ENREF_4)]. The study used a non-standard histological method (ink blots) followed by DTI of the un-fixed tissue. This study has a number of important limitations: (i) the unconventional histological approach; (ii) myolaminar orientation cannot be measured directly on a cut-surface (as discussed in the main manuscript); (iii) it uses this indirect approach to get their ground-truth measure of the orthogonal system, i.e. it uses a component taken from DTI (**e_1_^DTI^**) in assessment of the accuracy of **e_3_^DTI^**; (iv) it uses a multistep process with assumptions made at each at each step which may accumulate error; and, (v) it assumes small α’’ which may not be correct in all myocardial regions [[5](#_ENREF_5)].

In a comparison of DTI and histological measurement of laminar orientation Kung et al[[6](#_ENREF_6)] measured the difference between histologically and DTI determined sheetlet angles as 8° ± 27° and they concluded that this was sufficient evidence that myolaminar orientations measured by the DTI secondary and tertiary eigenvectors correspond to the myolaminar orientations observed in histology. As such the conclusions of Kung et al. (2011)[[6](#_ENREF_6)] are more positive about DTI measurement of laminar orientation than our conclusions. α’ and **e_1_^DTI^** were estimated based on the assumption of low α’’ from [[7](#_ENREF_7)]]). The study is carefully planned and methodical but is based on a preconceived literature based model of cardiac structure (as discussed in detail above). This approach is described in the paper as a direct histological method, but it is our view that it is indirect, and indeed the authors[[6](#_ENREF_6)] discuss histological reconstruction limitations at length. Although there are justifications for the approach, the effect of cumulative errors is not quantifiable. There will be error associated with: (i) the cutting and selection of the tangential-longitudinal plane; (ii) differing values of α’’ in the selected tangential-longitudinal plane (i.e. not all myocytes will have α’’ = 0° in the section); (iii) differing α’ and hence cross myocyte direction within the images of the tangential-longitudinal plane, leading to section selection difficulty; (iv) cutting the sections parallel to the cross myocyte direction; and, (v) fiducial marker based registration of the location back to the DTI after all these processing steps. The overall bias and variation between DTI and histology, combining all ROI studies, and the comparison of two sets of estimated standard sheetlet angles from histology to DTI, was found to be 8±27° (mean±SD). We find using a 3D approach that variation and error of **e_3_^DTI^** differs substantially according to cardiac region. The best performance of DTI was 22±14° but when summed over these regions is ~30±20° for DTI (and this changes little with changing DTI parameters).

**Eigenvalue Comparison**

There are greater grounds for sorting the ST local myocyte eigenvector from the ST sheetlet eigenvector, than there are grounds for sorting of the DTI local myocyte eigenvector from the DTI sheetlet eigenvector. ST has large magnitude differences between λ_1_ and λ_2_ and between λ_2_ and λ_3_. DTI, however, has moderate magnitude differences between λ_1_ and λ_2_ and small magnitude differences between λ_2_ and λ_3_. Good eigenvalue separation is a prerequisite for confidence in the assignment of the myocardial structural orientations, but good eigenvector separation does not, in itself, show that the orientation measurements are accurate.

Eigenvector misassignment, as defined in the main manuscript, is the assignment of an eigenvector to the incorrect structural feature due to imaging noise and small difference in eigenvalue magnitudes. Each tensor eigenvector has a corresponding eigenvalue, and the eigenvectors are ordered according to the size of the eigenvalues. Since the tensors are positive definite all the eigenvalues are positive. Sorting is simple and absolute (except in the case of identical values which is rare) and the eigenvalues cannot be missorted in a mathematical sense. However, there are negative implications for accurate measurement if there is little difference between the voxel-wise ratio of eigenvalues (although conversely, a large difference in eigenvalues is not necessarily evidence of the good performance of an imaging method). When the magnitudes of eigenvalues are close, a small variation in regional imaging noise between studies will result in many voxels having myolaminar normal orientations flipping from one orthogonal direction, to a second orthogonal direction, i.e. flipping between **s** and **n**. This may explain some variation between successive **e_2_^DTI^** and **e_3_^DTI^** orientations with different imaging times, or other imaging parameters. However, the generally low sensitivity of imaging parameters suggests that this flipping of **s** and **n** between imaging experiments is insignificant. The more important consideration with respect to the small differences between the voxel-wise ratio of eigenvalues is that it is evidence of the underlying complexity of tissue diffusion processes. Within an individual voxel diffusion is probably affected by the following structural features: (i) myocyte orientation; (ii) myocyte connectivity; (iii) myocyte density; (iv) capillary/blood vessel (BV) orientation; (v) capillary/BV connectivity; (vi) capillary/BV density; (vii) laminar/cleavage plane orientation; (viii) laminar/cleavage plane connectivity; and, (ix) laminar/cleavage plane density. This list is not exhaustive, but it is likely these are structures that must influence diffusion. There are other structural features which are likely to influence diffusion such as collagen orientation/density and lymphatics. Each of these structural influences operates at different spatial scales and restricts diffusion to different extents contributing to the non-monogaussian nature of myocardial image voxel diffusion. In DTI these factors are lumped together into a model which treats them as a single space with monogaussian diffusion[[8](#_ENREF_8)]. These structural factors together with limitations in the imaging model and imaging noise will have a local effect on each voxel dependent on their complex local admixing. It is not surprising that for DTI in fixed myocardium, under the range of imaging parameters explored that a substantial proportion of voxels λ_3_^DTI^ (and therefore **e_3_^DTI^**) may be associated with a direction strongly influenced by factors other than the laminar orientation. The principal myocyte direction is a strong structural feature at the microscopic scale, and as many other structural features are aligned to this direction (myofibrils, blood vessels), this orientation is more robust against mis-assignment, and hence there is a greater median difference between λ_1_^DTI^ and λ_2_^DTI^ than between λ_2_^DTI^ and λ_3_^DTI^. There was also evidence of this in the septal ROI for ST, but this was limited to 9.3% of septal voxels.

**REFERENCES**

1. Gilbert SH, Benoist D, Benson AP, White E, Tanner SF, Holden AV, Dobrzynski H, Bernus O, Radjenovic A: **Visualization and quantification of whole rat heart laminar structure using high-spatial resolution contrast-enhanced MRI.** *American Journal of Physiology-Heart and Circulatory Physiology* 2012, **302:**H287-H298.

2. Fisher R: **Dispersion on a Sphere.** *Proceedings of the Royal Society of London Series A, Mathematical and Physical Sciences* 1953, **217:**295-305.

3. Hales PW, Burton RA, Bollensdorff C, Mason F, Bishop M, Gavaghan D, Kohl P, Schneider JE: **Progressive changes in T(1), T(2) and left-ventricular histo-architecture in the fixed and embedded rat heart.** *NMR Biomed* 2011, **24:**836-843.

4. Tseng WY, Wedeen VJ, Reese TG, Smith RN, Halpern EF: **Diffusion tensor MRI of myocardial fibers and sheets: correspondence with visible cut-face texture.** *J Magn Reson Imaging* 2003, **17:**31-42.

5. Geerts L, Bovendeerd P, Nicolay K, Arts T: **Characterization of the normal cardiac myofiber field in goat measured with MR-diffusion tensor imaging.** *Am J Physiol Heart Circ Physiol* 2002, **283:**H139-145.

6. Kung GL, Nguyen TC, Itoh A, Skare S, Ingels NB, Jr., Miller DC, Ennis DB: **The presence of two local myocardial sheet populations confirmed by diffusion tensor MRI and histological validation.** *J Magn Reson Imaging* 2011, **34:**1080-1091.

7. Streeter DD: **Vol. I. The heart. Section 2: The cardiovascular system: Gross morphology and fiber geometry of the heart.** In *Handbook of Physiology Volume* 1. Edited by M. BR: Am. Physiol. Soc. Bethesda, MD; 1979: 61-112

8. Hsu EW, Buckley DL, Bui JD, Blackband SJ, Forder JR: **Two-component diffusion tensor MRI of isolated perfused hearts.** *Magn Reson Med* 2001, **45:**1039-1045.

**FIGURE DS1 (Additional file 4). Visualization of the difference between ST and DTI putative sheetlet-in-plane orientation vectors and angles**. **A** - angle between the DTI (6-direction) and ST putative sheetlet in-plane vectors, which are colored according to the 0º to +90º scale shown. **B** – the ST and DTI putative sheetlet in-plane elevation (β’) and sheetlet in-plane transverse(β’’) angle maps, which are colored according to the -90º to +90º scale shown. DTI: Scan #1, 6-direction, b = 1000 s/mm^2^; ST: Scan #8, DTW = 3, STW = 3. FLASH: fast low angle shot; ST: structure tensor of FLASH data; DTI: diffusion tensor magnetic resonance imaging; DTW: derivative template width STW: smoothing template width. The symbols for vectors and derived angles are defined in primary manuscript Table 2.

**FIGURE DS2 (Additional file 6). The ST and DTI putative sheetlet-normal angles are compared for 5 rat hearts.** The **v_3_^ST^** and **e_3_^DTI^** transverse (β’’) angle maps of an equatorial short-axis slice are colored according to the -90º to +90º scale. Regions of similar and differing laminar normal orientation are shown in the magenta and black boxes respectively. The transmural orange line on the FLASH images indicates the transmural span quantified in Fig. 14. DTI: Scan #1, 6-direction, b = 1000 s/mm^2^; ST: Scan #8, DTW = 3, STW = 3. FLASH: fast low angle shot; ST: structure tensor of FLASH data; DTI: diffusion tensor magnetic resonance imaging; DTW: derivative template width STW: smoothing template width. The symbols for vectors and derived angles are defined in primary manuscript Table 2. The associated angle maps for the **v_3_^ST^** and **e_3_^DTI^** elevation (β’) angle are in main manuscript Fig. 9.

**FIGURE DS3 (Additional file 7). Equatorial lateral ROI distributions of angle differences between the ST and DTI vectors/orientation angles.** **A** – deviation angles |∠**v_3_^ST^e_3_^DTI^**| and |∠**v_2_^ST^e_2_^DTI^**| are shown, alongside the corresponding distributions of |∠β’**v_3_^ST^**β’**e_3_^DTI^**|,|∠β’’**v_3_^ST^**β’’**e_3_^DTI^**|,|∠β’**v_2_^ST^**β’**e_2_^DTI^**|,|∠β’’**v_2_^ST^**β’’**e_2_^DTI^**|. **B** – deviation angles |∠**v_1_^ST^e_1_^DTI^**| and |∠**v_1_^ST^e_1_^DTI^**| are shown, alongside the corresponding distributions of |∠α’**v_1_^ST^**α’**e_1_^DTI^**|, |∠α’’**v_1_^ST^**α’’**e_1_^DTI^**|, |∠α’**v_1_^ST^**α’**e_1_^DTI^**|, |∠α’’**v_1_^ST^**α’’**e_1_^DTI^**|. DTI: Scan #1, 6-direction, b = 1000 s/mm^2^; ST: Scan #8; DTW = 3; STW = 3. FLASH: fast low angle shot; ST: structure tensor of FLASH data; DTI: diffusion tensor magnetic resonance imaging; DTW: derivative template width STW: smoothing template width; MAD: median absolute deviation; ROI: region(s) of interest. The symbols for vectors and derived angles are defined in primary manuscript Table 2. The corresponding distributions for the equatorial septal ROI are in primary manuscript Fig. 10.

**FIGURE DS4 (Additional file 8).** **Differences between ST and DTI vary depending on cardiac location and are stable over time.** Results are presented by region (lateral, septal) showing the deviation between the ST and the corresponding DTI eigenvector orientations pairs (of **v_1_^ST^e_1_^DTI^** , **v_2_^ST^e_2_^DTI^**  and **v_3_^ST^e_3_^DTI^** ), and the difference between the associated vector elevation and transverse angles. Side **A** (left) of each histogram are angles from comparison of ST to a DTI image taken in the 2 hours BEFORE the FLASH (Scan #7). Side **B** are from comparison of ST to a DTI image taken in the 2 hours AFTER the FLASH (Scan #9). DTI: 6-direction, b = 1000 s/mm^2^; ST: Scan #8, DTW = 3, STW = 3. FLASH: fast low angle shot; ST: structure tensor of FLASH data; DTI: diffusion tensor magnetic resonance imaging; DTW: derivative template width STW: smoothing template width. The symbols for vectors and derived angles are defined in primary manuscript Table 2. The corresponding distributions for the lateral and septal ROI are in main manuscript Fig. 11.

**FIGURE DS5 (Additional file 5). Summary sensitivity analysis data, referenced to the ground truth, for all scans for all ROI.** The mean and standard deviation of |∠**[v_3_^ST^n^FI^**| or |∠**[e_3_^DTI^n^FI^**| are shown for the lateral, septal, anterior and posterior ROI. The number following the # is the scan number as defined in primary manuscript Table 1. ^a^ – scan # 8, T1W FLASH scan with processing parameters STW = 3, DTW = 3. ^b^ – scan # 8, FLASH T1W FLASH scan with processing parameters STW = 5, DTW = 5. FLASH: fast low angle shot; ST: structure tensor of FLASH data; DTI: diffusion tensor magnetic resonance imaging; DTW: derivative template width STW: smoothing template width. The symbols for vectors and derived angles are defined in primary manuscript Table 2.

**FIGURE DS6 (Additional file 10). Sensitivity of the deviation between v_3_^ST^** or **e_3_^DTI^ and n^FI^ to imaging/image processing parameters.** This was explored in the from the four ROI (lateral, septal, anterior, posterior), showing the mean and standard deviation of |∠**[v_3_^ST^n^FI^**| or |∠**[e_3_^DTI^n^FI^**|. **A** - sensitivity of **e_3_^DTI^** to time post-fixation from 2 to 71 hours. **B** - sensitivity of **e_3_^DTI^** to b-value from 500 to 2500 s/mm^2^. **C** - sensitivity of **e_3_^DTI^** to number of diffusion directions from 6 to 12 directions. **D** – sensitivity of **v_3_^ST^** to the DTW and STW. ST: Scan #8. FLASH: fast low angle shot; ST: structure tensor of FLASH data; DTI: diffusion tensor magnetic resonance imaging; DTW: derivative template width STW: smoothing template width. The symbols for vectors and derived angles are defined in primary manuscript Table 2.

**TABLE DS1**. **Summary of the sensitivity of ST and DTI measurements to imaging and image-processing parameters.** Parameters include DTI sensitivity to time post-fixation, b-value and number of directions and ST sensitivity to time post-fixation, diffusion template width, and smoothing template width. The summary sensitivity values are the range of sensitivity of the **m**/**s**/**n** orientation over the parameter range explored. This gives an idea of total sensitivity to the parameter explored, but does not give the optimal parameter value, which is given in the relevant figures and text. **§ -** ST_2_ values are referenced to ST_1_ values but there is no appropriate FI value against which these data are referenced. **†** - the mean-difference between the measured vector and the reference vector was determined for each imaging parameter value, and the absolute value of the maximum sensitivity reported is the maximum divergence in mean-difference across the parameter range explored.

**TABLE DS1**

| **Vector** | **Reference vector** | **\|Sensitivity to parameter (change in mean angle with respect to the reference angle over parameter range)†\|**  **(°)** | **Optimum parameter (in units of parameter)** |
| --- | --- | --- | --- |
| **DIFFUSION TENSOR (DTI)** | | |  |
| **Sensitivity to time between 3:50 and 72:45 (h:min)** | | | |
| **e_1_^DTI^** | **v_1_^ST^** | **1.5°** | **3:50 (h:min)** |
| **e_2_^DTI^** | **v_2_^ST^** | **0.9°** | **46:57 (h:min)** |
| **e_3_^DTI^** | **v_3_^ST^** | **2.3°** | **46:57 (h:min)** |
| **e_3_^DTI^** | **n^FI^** | **3.4°** | **21:08 (h:min)** |
| **Sensitivity to b-value between 500 s/mm^2^ and 2500 s/mm^2^** | | | |
| **e_1_^DTI^** | **v_1_^ST^** | **4.0°** | **1000 s/mm^2^** |
| **e_2_^DTI^** | **v_2_^ST^** | **8.3°** | **1000 s/mm^2^** |
| **e_3_^DTI^** | **v_3_^ST^** | **7.8°** | **1000 s/mm^2^** |
| **e_3_^DTI^** | **n^FI^** | **3.1°** | **1000 s/mm^2^** |
| **Sensitivity to number of diffusion directions between 6 and 12** | | | |
| **e_1_^DTI^** | **v_1_^ST^** | **0.3°** | **12** |
| **e_2_^DTI^** | **v_2_^ST^** | **4.2°** | **12** |
| **e_3_^DTI^** | **v_3_^ST^** | **3.9°** | **12** |
| **e_3_^DTI^** | **n^FI^** | **0.9°** | **12** |
| **STRUCTURE TENSOR (ST)** | | | |
| **Sensitivity to time between 3:50 and 72:45 (h:min)** | | | |
| **v_1_^ST^** | **prior v_1_^ST^** | **4.9°** | **§** |
| **v_2_^ST^** | **prior v_2_^ST^** | **7.1°** | **§** |
| **v_3_^ST^** | **prior v_3_^ST^** | **3.6°** | **§** |
| **Sensitivity to STW and DTW (STW&DTW = 3 OR STW&DTW = 5)** | | | |
| **v_3_^ST^** | **n^FI^** | **0.6°** | **STW=3, DTW=3** |

1. [↑](#footnote-ref-1)
