# Supplementary material for: Comparison of diffusion tensor imaging by cardiovascular magnetic resonance and gadolinium enhanced 3D image intensity approaches to investigation of structural anisotropy in explanted rat hearts
Source: J Cardiovasc Magn Reson. 2015 Apr 29;17(1):31. doi: 10.1186/s12968-015-0129-x (PMC4414435; doi:10.1186/s12968-015-0129-x)
Supplement: Additional file 3: Figure DS1. — Visualization of the difference between ST and DTI putative sheetlet-in-plane orientation vectors and angles. A - angle between the DTI (6-direction) and ST putative sheetlet in-plane vectors, which are colored according to the 0° to +90° scale shown. B - the ST and DTI putative sheetlet in-plane elevation (β’) and sheetlet in-plane transverse(β’’) angle maps, which are colored according to the -90° to +90° scale shown. DTI: Scan #1, 6-direction, b = 1000 s/mm2; ST: Scan #8, DTW = 3, STW = 3. FLASH: fast low angle shot; ST: structure tensor of FLASH data; DTI: diffusion tensor magnetic resonance imaging; DTW: derivative template width STW: smoothing template width. The symbols for vectors and derived angles are defined in Table 2. [file 12968_2015_129_MOESM3_ESM.pptx]

## Slide 1
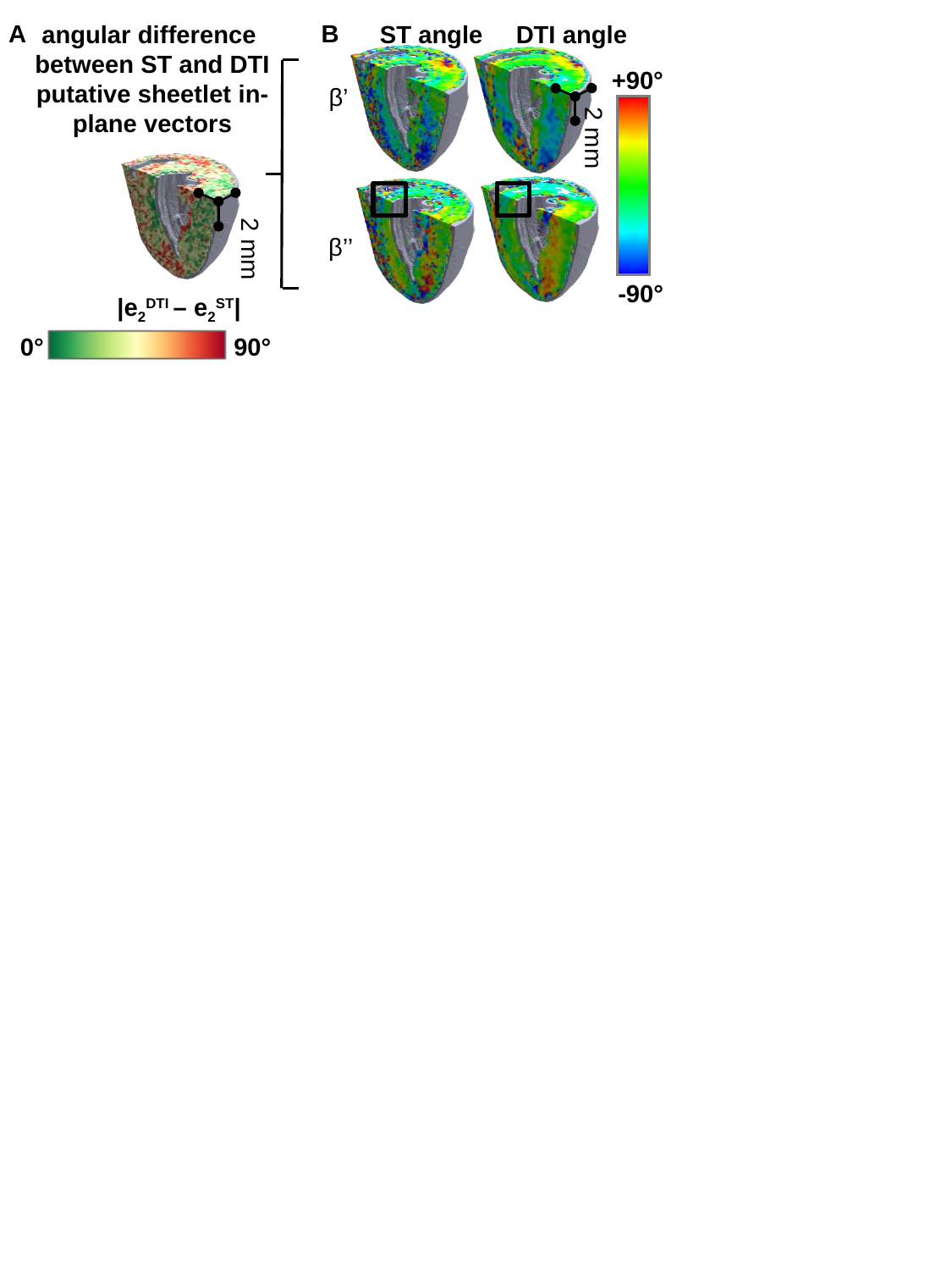

angular difference
between ST and DTI
putative sheetlet in-plane vectors
A
B
ST angle DTI angle
+90°
-90°
β’
β’’
2 mm
2 mm
90°
0°
|e2DTI – e2ST|
