# Supplementary material for: Comparison of diffusion tensor imaging by cardiovascular magnetic resonance and gadolinium enhanced 3D image intensity approaches to investigation of structural anisotropy in explanted rat hearts
Source: J Cardiovasc Magn Reson. 2015 Apr 29;17(1):31. doi: 10.1186/s12968-015-0129-x (PMC4414435; doi:10.1186/s12968-015-0129-x)
Supplement: Additional file 5: Figure DS5. — Summary sensitivity analysis data, referenced to the ground truth, for all scans for all ROI. The mean and standard deviation of |∠[v 3 ST n FI| or |∠[e 3 DTI n FI| are shown for the lateral, septal, anterior and posterior ROI. The number following the # is the scan number as defined in Table 1. a – scan # 8, T1W FLASH scan with processing parameters STW = 3, DTW = 3. b – scan # 8, FLASH T1W FLASH scan with processing parameters STW = 5, DTW = 5. FLASH: fast low angle shot; ST: structure tensor of FLASH data; DTI: diffusion tensor magnetic resonance imaging; DTW: derivative template width STW: smoothing template width. The symbols for vectors and derived angles are defined in Table 2. [file 12968_2015_129_MOESM5_ESM.pptx]

## Slide 1
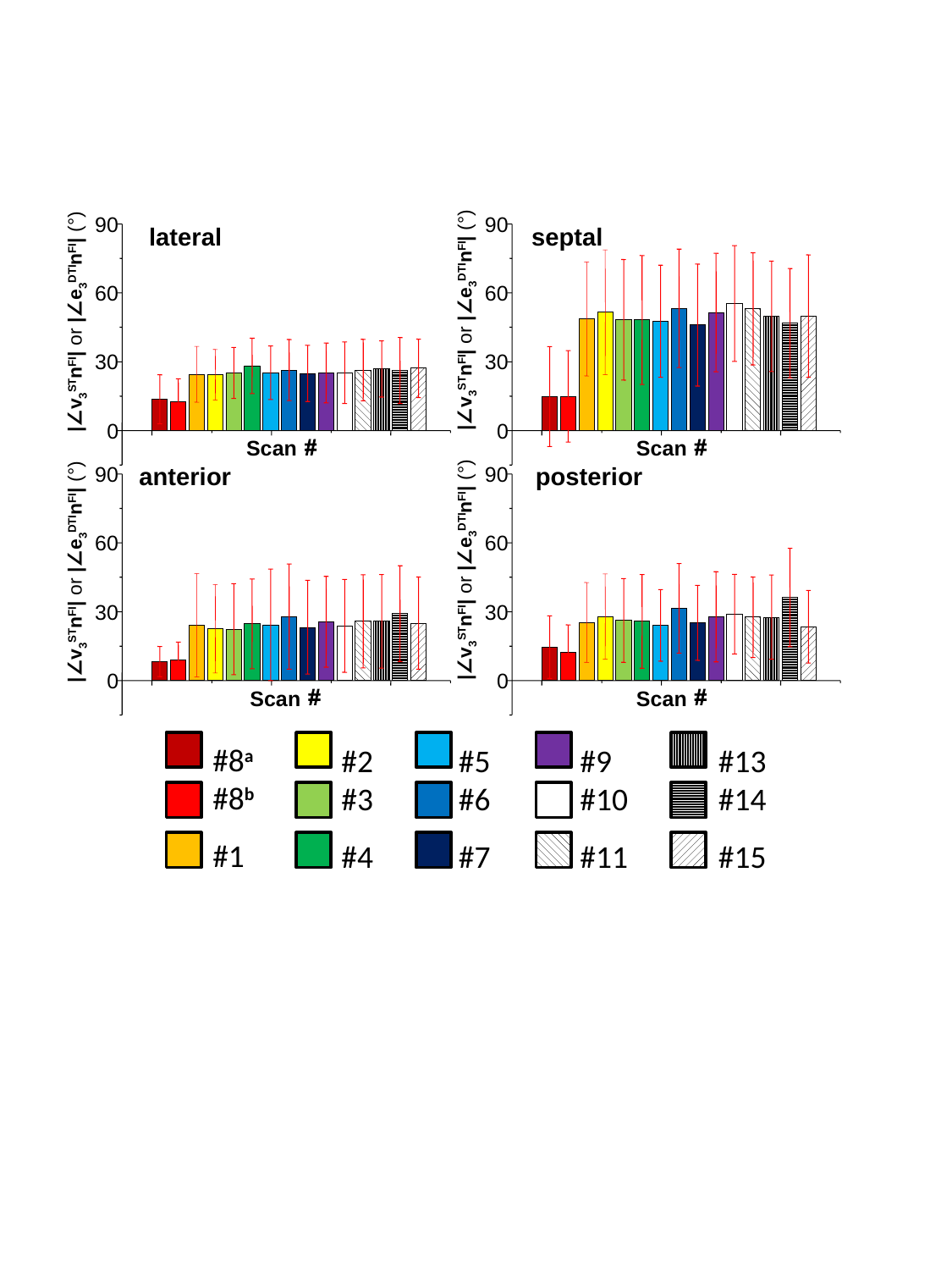

90
60
30
0
 #
Scan
90
60
30
0
 #
Scan
lateral
septal
90
60
30
0
 #
Scan
90
60
30
0
 #
Scan
anterior
posterior
#8a
#8b
#1
#2
#3
#4
#5
#6
#7
#9
#10
#11
#13
#14
#15
|∠v3STnFI| or |∠e3DTInFI| (°)
|∠v3STnFI| or |∠e3DTInFI| (°)
|∠v3STnFI| or |∠e3DTInFI| (°)
|∠v3STnFI| or |∠e3DTInFI| (°)
