# Supplementary figures and images for: Comparison of diffusion tensor imaging by cardiovascular magnetic resonance and gadolinium enhanced 3D image intensity approaches to investigation of structural anisotropy in explanted rat hearts
Source: J Cardiovasc Magn Reson. 2015 Apr 29;17(1):31. doi: 10.1186/s12968-015-0129-x (PMC4414435; doi:10.1186/s12968-015-0129-x)

## Slide 1
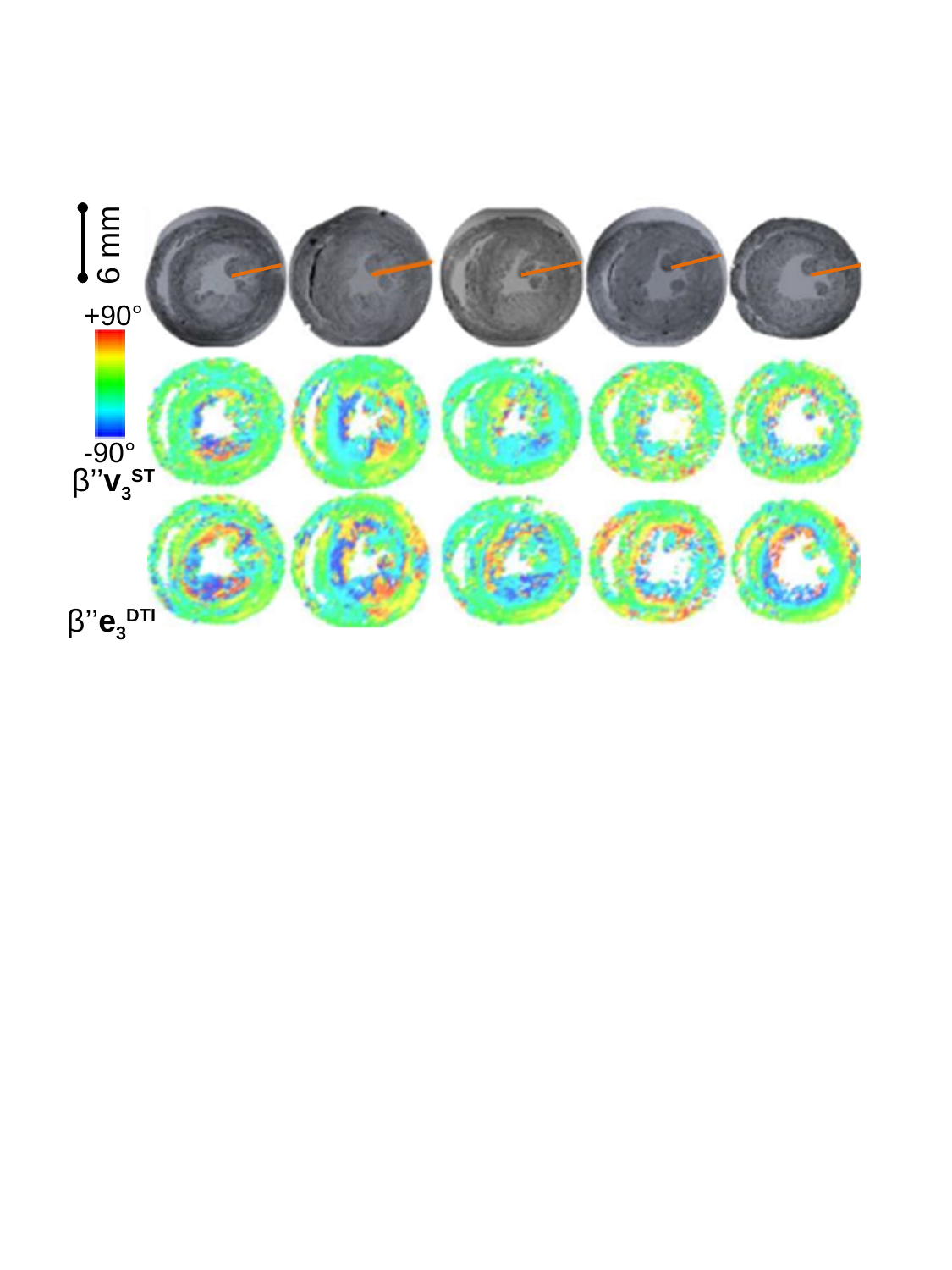

6 mm
+90°
-90°
β’’v3ST
β’’e3DTI

Supplement: Additional file 6: Figure DS2. — The ST and DTI putative sheetlet-normal angles are compared for 5 rat hearts. The v 3 ST and e 3 DTI transverse (β’’) angle maps of an equatorial short-axis slice are colored according to the -90° to +90° scale. Regions of similar and differing laminar normal orientation are shown in the magenta and black boxes respectively. The transmural orange line on the FLASH images indicates the transmural span quantified in Figure 14. DTI: Scan #1, 6-direction, b = 1000 s/mm2; ST: Scan #8, DTW = 3, STW = 3. FLASH: fast low angle shot; ST: structure tensor of FLASH data; DTI: diffusion tensor magnetic resonance imaging; DTW: derivative template width STW: smoothing template width. The symbols for vectors and derived angles are defined in Table 2. The associated angle maps for the v 3 ST and e 3 DTI elevation (β’) angle are in Figure 9. [file 12968_2015_129_MOESM6_ESM.pptx]
