# Supplementary material for: Comparison of diffusion tensor imaging by cardiovascular magnetic resonance and gadolinium enhanced 3D image intensity approaches to investigation of structural anisotropy in explanted rat hearts
Source: J Cardiovasc Magn Reson. 2015 Apr 29;17(1):31. doi: 10.1186/s12968-015-0129-x (PMC4414435; doi:10.1186/s12968-015-0129-x)
Supplement: Additional file 7: Figure DS3. — Equatorial lateral ROI distributions of angle differences between the ST and DTI vectors/orientation angles. A – deviation angles |∠v 3 ST e 3 DTI| and |∠v 2 ST e 2 DTI| are shown, alongside the corresponding distributions of |∠β’v 3 STβ’e 3 DTI|, |∠β”v 3 STβ”e 3 DTI|, |∠β’v 2 STβ’e 2 DTI|, |∠β”v 2 STβ”e 2 DTI|. B – deviation angles |∠v 1 ST e 1 DTI| and |∠v 1 ST e 1 DTI| are shown, alongside the corresponding distributions of |∠α’v 1 STα’e 1 DTI|, |∠α”v 1 STα”e 1 DTI|, |∠α’v 1 STα’e 1 DTI|, |∠α”v 1 STα”e 1 DTI|. DTI: Scan #1, 6-direction, b = 1000 s/mm2; ST: Scan #8; DTW = 3; STW = 3. FLASH: fast low angle shot; ST: structure tensor of FLASH data; DTI: diffusion tensor magnetic resonance imaging; DTW: derivative template width STW: smoothing template width; MAD: median absolute deviation; ROI: region(s) of interest. The symbols for vectors and derived angles are defined in Table 2. The corresponding distributions for the equatorial septal ROI are in Figure 10. [file 12968_2015_129_MOESM7_ESM.pptx]

## Slide 1
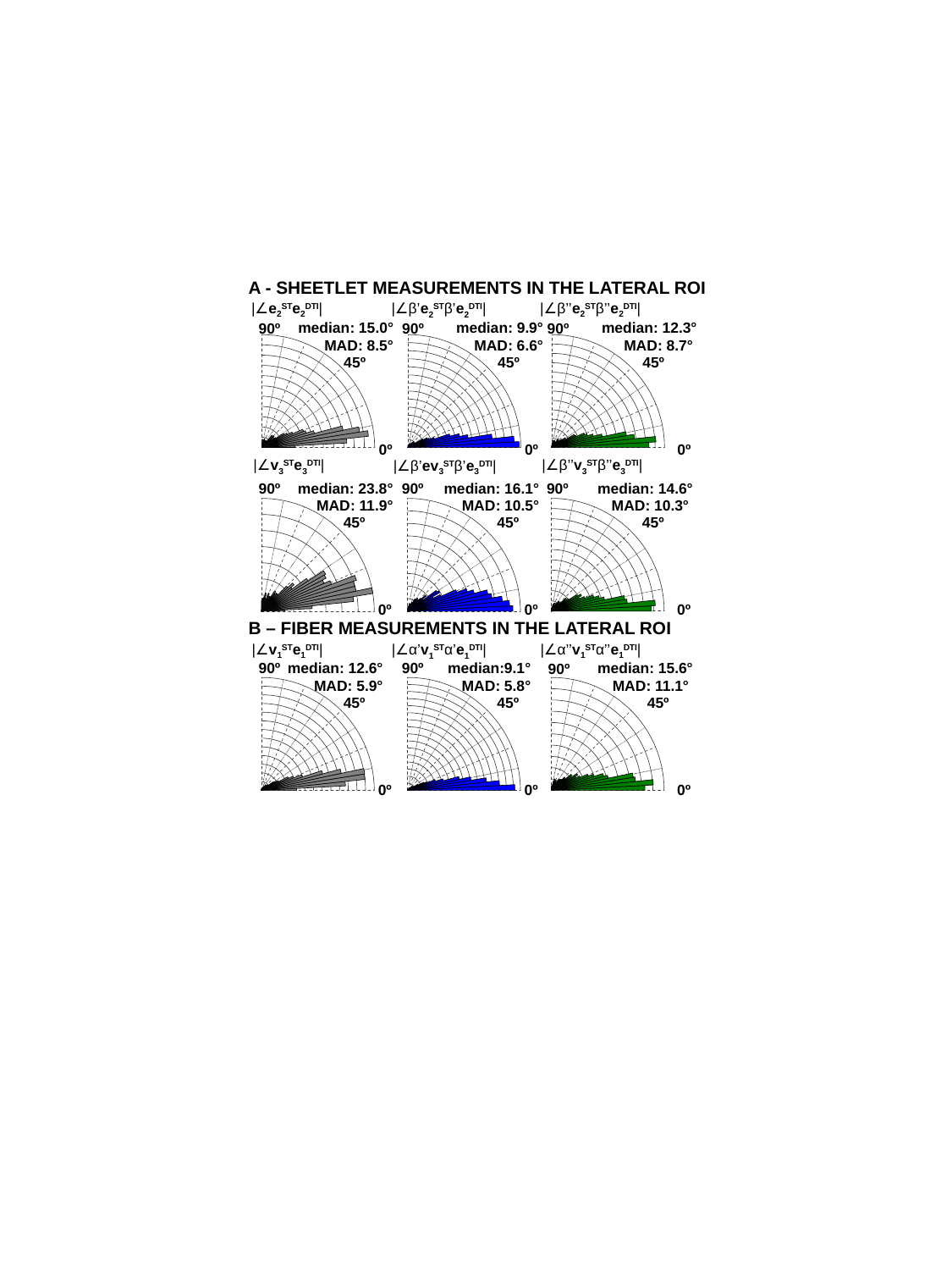

A - SHEETLET MEASUREMENTS IN THE LATERAL ROI
|∠e2STe2DTI|
|∠β’’e2STβ’’e2DTI|
|∠β’e2STβ’e2DTI|
|∠v3STe3DTI|
|∠β’’v3STβ’’e3DTI|
|∠β’ev3STβ’e3DTI|
B – FIBER MEASUREMENTS IN THE LATERAL ROI
|∠v1STe1DTI|
|∠α’’v1STα’’e1DTI|
|∠α’v1STα’e1DTI|
90º
median: 15.0°
MAD: 8.5°
90º
median: 9.9°
MAD: 6.6°
90º
median: 12.3°
MAD: 8.7°
45º
45º
45º
0º
0º
0º
90º
median: 23.8°
MAD: 11.9°
90º
median: 16.1°
MAD: 10.5°
90º
median: 14.6°
MAD: 10.3°
45º
45º
45º
0º
0º
0º
90º
median: 12.6°
MAD: 5.9°
90º
median:9.1°
MAD: 5.8°
median: 15.6°
MAD: 11.1°
90º
45º
45º
45º
0º
0º
0º
