# Supplementary material for: Comparison of diffusion tensor imaging by cardiovascular magnetic resonance and gadolinium enhanced 3D image intensity approaches to investigation of structural anisotropy in explanted rat hearts
Source: J Cardiovasc Magn Reson. 2015 Apr 29;17(1):31. doi: 10.1186/s12968-015-0129-x (PMC4414435; doi:10.1186/s12968-015-0129-x)
Supplement: Additional file 8: Figure DS4. — Differences between ST and DTI vary depending on cardiac location and are stable over time. Results are presented by region (lateral, septal) showing the deviation between the ST and the corresponding DTI eigenvector orientations pairs (of v 1 ST e 1 DTI , v 2 ST e 2 DTI and v 3 ST e 3 DTI), and the difference between the associated vector elevation and transverse angles. Side A (left) of each histogram are angles from comparison of ST to a DTI image taken in the 2 hours BEFORE the FLASH (Scan #7). Side B are from comparison of ST to a DTI image taken in the 2 hours AFTER the FLASH (Scan #9). DTI: 6-direction, b = 1000 s/mm2; ST: Scan #8, DTW = 3, STW = 3. FLASH: fast low angle shot; ST: structure tensor of FLASH data; DTI: diffusion tensor magnetic resonance imaging; DTW: derivative template width STW: smoothing template width. The symbols for vectors and derived angles are defined in Table 2. The corresponding distributions for the lateral and septal ROI are in Figure 11. [file 12968_2015_129_MOESM8_ESM.pptx]

## Slide 1
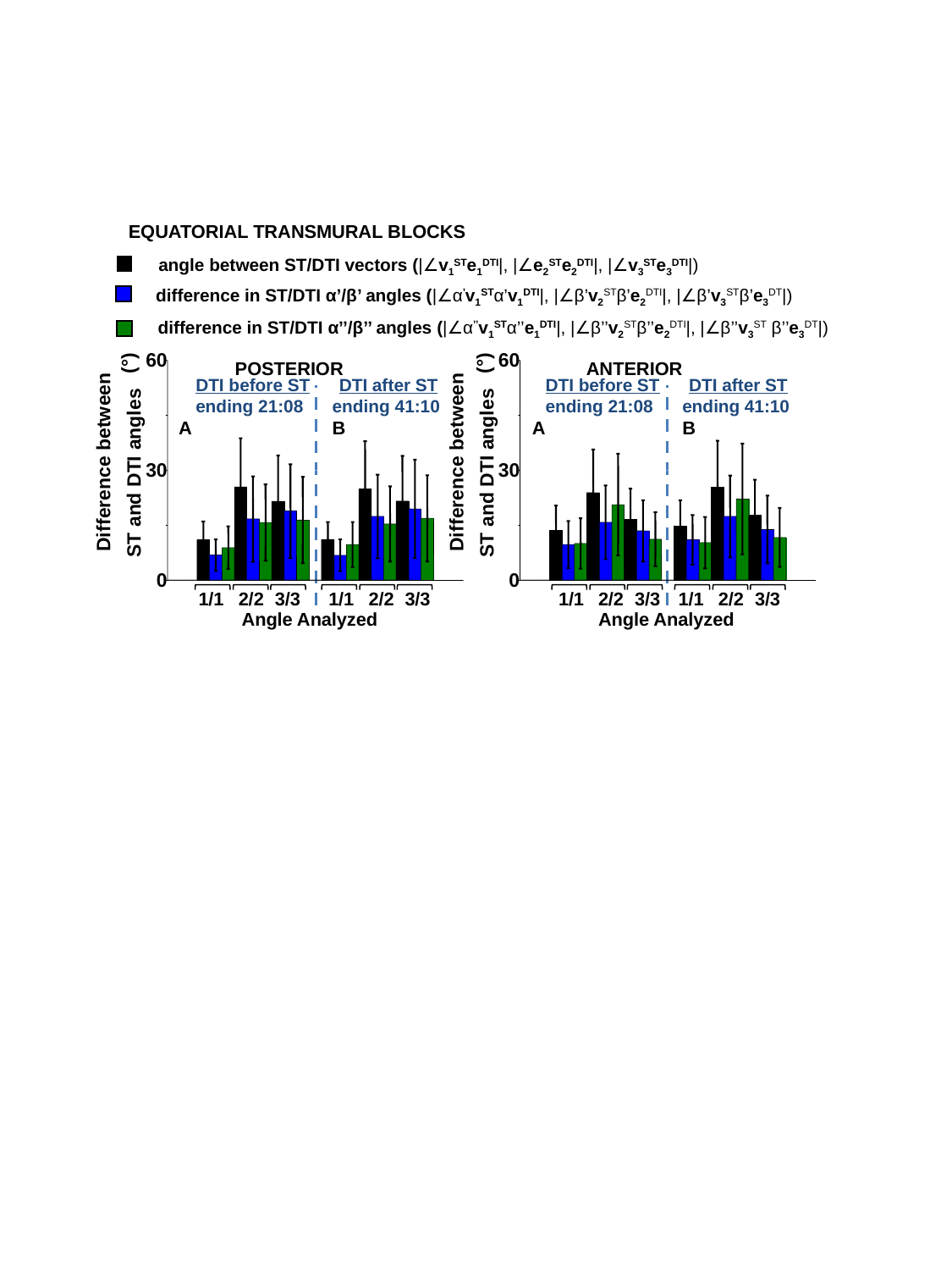

EQUATORIAL TRANSMURAL BLOCKS
angle between ST/DTI vectors (|∠v1STe1DTI|, |∠e2STe2DTI|, |∠v3STe3DTI|)
difference in ST/DTI α’/β’ angles (|∠α’v1STα’v1DTI|, |∠β’v2STβ’e2DTI|, |∠β’v3STβ’e3DT|)
difference in ST/DTI α’’/β’’ angles (|∠α’’v1STα’’e1DTI|, |∠β’’v2STβ’’e2DTI|, |∠β’’v3ST β’’e3DT|)
60
 (°)
Difference between
30
 ST and DTI angles
0
60
 (°)
Difference between
30
 ST and DTI angles
0
POSTERIOR
ANTERIOR
DTI before ST DTI after ST
ending 21:08 ending 41:10
DTI before ST DTI after ST
ending 21:08 ending 41:10
A
B
A
B
1/1
2/2
3/3
1/1
2/2
3/3
1/1
2/2
3/3
1/1
2/2
3/3
Angle Analyzed
Angle Analyzed
