# Supplementary material for: Comparison of diffusion tensor imaging by cardiovascular magnetic resonance and gadolinium enhanced 3D image intensity approaches to investigation of structural anisotropy in explanted rat hearts
Source: J Cardiovasc Magn Reson. 2015 Apr 29;17(1):31. doi: 10.1186/s12968-015-0129-x (PMC4414435; doi:10.1186/s12968-015-0129-x)
Supplement: Additional file 10: Figure DS6. — Sensitivity of the deviation between v 3 ST or e 3 DTI and n FI to imaging/image processing parameters. This was explored in the from the four ROI (lateral, septal, anterior, posterior), showing the mean and standard deviation of |∠[v 3 ST n FI| or |∠[e 3 DTI n FI|. A – sensitivity of e 3 DTI to time post-fixation from 2 to 71 hours. B – sensitivity of e 3 DTI to b-value from 500 to 2500 s/mm2. C – sensitivity of e 3 DTI to number of diffusion directions from 6 to 12 directions. D – sensitivity of v 3 ST to the DTW and STW. ST: Scan #8. FLASH: fast low angle shot; ST: structure tensor of FLASH data; DTI: diffusion tensor magnetic resonance imaging; DTW: derivative template width STW: smoothing template width. The symbols for vectors and derived angles are defined in Table 2. [file 12968_2015_129_MOESM10_ESM.pptx]

## Slide 1
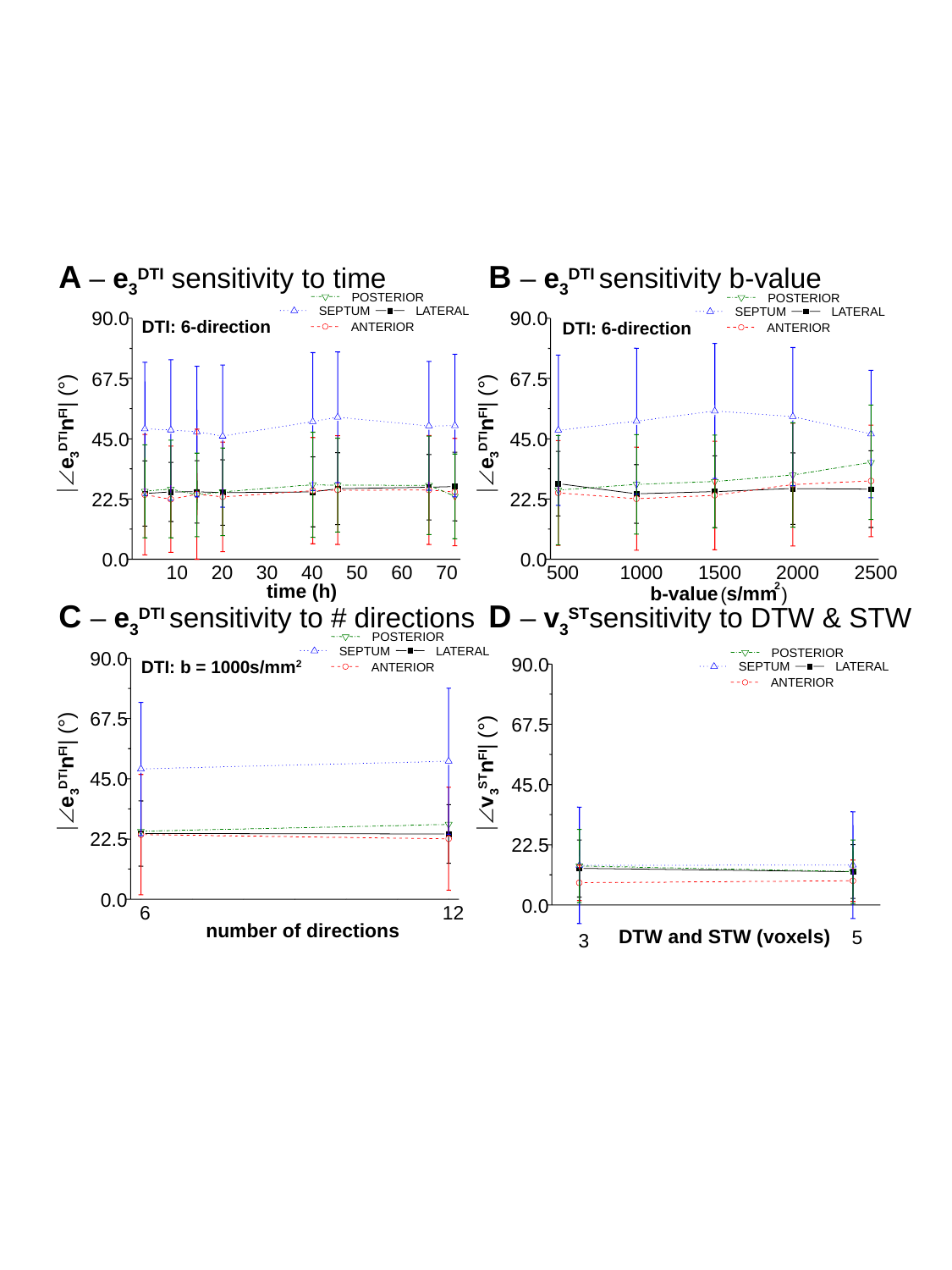

A – e3DTI sensitivity to time
B – e3DTI sensitivity b-value
POSTERIOR
SEPTUM
LATERAL
ANTERIOR
POSTERIOR
SEPTUM
LATERAL
ANTERIOR
DTI: 6-direction
DTI: 6-direction
C – e3DTI sensitivity to # directions
D – v3STsensitivity to DTW & STW
POSTERIOR
SEPTUM
LATERAL
ANTERIOR
POSTERIOR
SEPTUM
LATERAL
ANTERIOR
DTI: b = 1000s/mm2
90.0
67.5
45.0
22.5
0.0
10
20
30
40
50
60
70
time (h)
90.0
67.5
45.0
22.5
0.0
500
1000
1500
2000
2500
2
b-value
s/mm
(
)
|Ðe3DTInFI| (°)
|Ðe3DTInFI| (°)
90.0
67.5
45.0
22.5
0.0
6
12
number of directions
90.0
67.5
45.0
22.5
0.0
5
3
DTW and STW (voxels)
|Ðe3DTInFI| (°)
|Ðv3STnFI| (°)
